# P53 represses pyrimidine catabolic gene *dihydropyrimidine dehydrogenase* (*DPYD*) expression in response to thymidylate synthase (TS) targeting

**DOI:** 10.1038/s41598-017-09859-x

**Published:** 2017-08-29

**Authors:** Prashanth Gokare, Niklas K. Finnberg, Phillip H. Abbosh, Jenny Dai, Maureen E. Murphy, Wafik S. El-Deiry

**Affiliations:** 1 Laboratory of Translational Oncology and Experimental Cancer Therapeutics, Department of Hematology/Oncology and Molecular Therapeutics Program, Fox Chase Cancer Center, Philadelphia, PA 19111 USA; 2 Penn State Hershey Cancer Institute, Penn State Hershey Medical Center, 500 University Dr, Hershey, PA 17033 USA; 3 Molecular and Cellular Oncogenesis Program, The Wistar Institute, Philadelphia, PA 19104 USA

**Keywords:** Cancer therapeutic resistance, Chemotherapy

## Abstract

Nucleotide metabolism in cancer cells can influence malignant behavior and intrinsic resistance to therapy. Here we describe p53-dependent control of the rate-limiting enzyme in the pyrimidine catabolic pathway, dihydropyrimidine dehydrogenase (DPYD) and its effect on pharmacokinetics of and response to 5-fluorouracil (5-FU). Using *in silico*/chromatin-immunoprecipitation (ChIP) analysis we identify a conserved p53 DNA-binding site (p53BS) downstream of the *DPYD* gene with increased p53 occupancy following 5-FU treatment of cells. Consequently, decrease in Histone H3K9AC and increase in H3K27me3 marks at the *DPYD* promoter are observed concomitantly with reduced expression of DPYD mRNA and protein in a p53-dependent manner. Mechanistic studies reveal inhibition of DPYD expression by p53 is augmented following thymidylate synthase (TS) inhibition and DPYD repression by p53 is dependent on DNA-dependent protein kinase (DNA-PK) and Ataxia telangiectasia mutated (ATM) signaling. *In-vivo*, liver specific Tp53 loss increases the conversion of 5-FU to 5-FUH_2_ in plasma and elicits a diminished 5-FU therapeutic response in a syngeneic colorectal tumor model consistent with increased DPYD-activity. Our data suggest that p53 plays an important role in controlling pyrimidine catabolism through repression of DPYD expression, following metabolic stress imposed by nucleotide imbalance. These findings have implications for the toxicity and efficacy of the cancer therapeutic 5-FU.

## Introduction

Dihydropyrimidine dehydrogenase (DPYD) is the initial rate-limiting enzyme of the pyrimidine catabolic pathway that catalyzes the reduction of the nucleotide bases uracil and thymine^1^. Although recent findings have implicated *DPYD* in epithelial-to-mesenchymal transition (EMT) of breast cancer cells^2^, most interest in DPYD has stemmed from its role in limiting the bioavailability of the chemotherapeutic anti-metabolite 5-fluorouracil (5-FU) that exerts its therapeutic activity at least partly through anabolic uptake^3–7^. Hepatic catabolism of 5-FU by DPYD is primarily responsible for the rate-limiting catabolic conversion to 5-fluoro-5,6-dihydrouracil (5-FUH_2_) which is a substrate in additional enzymatic steps resulting in the urinary excretion of fluorinated forms of beta-alanine and urea. Variable expression of DPYD and genetic polymorphisms in the *DPYD* gene have been linked to a high intra- and inter-patient variability in the plasma levels of 5-FU with associated toxicity and cancer drug resistance^8–10^. Subsequently, different pharmacological DPYD inhibitors such as gimeracil^11^ and eniluracil^12–14^ have been added to oral 5-FU formulations that currently are either approved for clinical practice or undergoing clinical trials in order to improve 5-FU bioavailability^15^.

The tumor suppressor gene *TP53* that encodes for the transcription factor p53 is mutated and/or inactivated in the majority of human cancers. Canonical p53 signaling involves induced transcription of genes involved in cell cycle arrest, DNA damage repair and programmed forms of cell death. However, it is becoming increasingly clear that p53 also modulates additional cellular processes such as metabolic pathways that can have a profound impact on cancer cell invasion and treatment refractoriness. Moreover, p53’s role as a transcriptional repressor may contribute to the biological phenotypes of its tumor suppressive action. In the context of 5-FU-based therapies, *TP53* mutation status has been correlated with treatment response and survival. Colorectal cancer patients with mutant *TP53* have a shorter overall survival as compared to patients with wild-type *TP53* ^16–18^. Indeed, colorectal cancer cells deficient for *TP53* and subjected to treatment with 5-FU in preclinical experiments are protected from cell death^19^. Interestingly, it is less clear if *TP53* status dictates the response to other DNA-damaging chemotherapies used for the treatment of colorectal cancer such as oxaliplatin and irinotecan that could indicate intrinsic differences in the p53 response between these chemotherapeutic agents^20, 21^.

In order to investigate how p53 selectively modulates the cellular response to 5-FU we performed an *in-silico* screen for p53 DNA-binding sites (p53BS) in the proximity of or within genes involved in nucleotide metabolism. By combining this analysis with chromatin immunoprecipitation (ChIP), expression analysis *in vitro* and *in vivo* we show that the expression of *DPYD* is negatively regulated by p53 in the context of inhibition of thymidylate synthase (TS). We show that this observation is correlated with increased relative levels of the 5-FUH_2_ catabolite and reduced tumor growth delay in mice lacking *TP53* in their livers following treatment with 5-FU. The data also indicate a role of the *TP53* codon R72P polymorphism in *DPYD* expression. Together, our current study provides novel insights into the role of p53 as a repressor of the key rate-limiting enzyme DPYD and indicates that p53 may function as a negative regulator of pyrimidine catabolism. Our results have implications for the toxicity of 5-FU as well as its efficacy in the treatment of cancer.

## Material and Methods

### Cell culture and treatments

Authenticated Cell lines were obtained from ATCC between the year 2011-12 and were within 20 passages when used for the experiments. HCT116-p53WT and HCT-116-p53^−/−^ cells were obtained from Bert Vogelstein at Johns Hopkins University. All cell lines were routinely tested for Mycoplasma for every 3 months by DAPI staining and PCR. Every 6 months STR profiling was performed for verification of cell line origin. HCT-116-p53WT and HCT-116-p53^−/−^ were cultured in McCoy’s 5 A media, H460 were cultured in RPMI1640. A549, U87MG, HT-1080 and R72P MEFs were cultured in DMEM. NHF cells were purchased from Coriell Institute for Medical Research (Camden, NJ, USA) and were grown in DMEM (15%PBS and non-essential amino acids). For the assessment of protein and mRNA expression, 5 × 10^5^ - 8 × 10^5^ cells were plated in 6-well plates (Corning) and treated for up to 24-hrs.

### Mice and treatments

Six to eight-week old male C57BL/6 J (wild-type), B6.129P2-Trp53tm1Brn/J (p53 ^l^°^xP/loxP^) and 129-Trp53tm1Tyj/J (p53^−/−^) and B6.Cg-Tg(Alb-cre)21Mgn/J (AlbCre) mice were purchased from Jackson Laboratory (Jackson Laboratory, ME). All mice were housed in a controlled environment with regard to light, temperature and humidity. Mice were euthanized through cervical dislocation following ketamine/xylazine anesthesia. All animal work was conducted based on the procedures and guidelines approved by the Penn State Hershey Medical Center Institutional Animal Care and Use Committee. 5-FU was injected IV at 100 mg/kg bolus for 4 hrs for analyzing p53 binding and for 6 hrs for DPYD western blotting experiments using frozen liver tissues. For plasma analysis of 5-FU and 5-FUH2, mice were given 150 mg/kg IV 5-FU or Vehicle (PBS) for 6 hrs and then a second dose of bolus 5-FU 150 mg/kg IV was administered to all the mice for 30 min after which about 300–500 micro-liters of blood were immediately collected by cardiac puncture and stored in −80 ^o^C for HPLC/MS analysis.

### Mouse syngeneic colorectal cancer model

A total of 1 × 10^6^ p53-Ras-Myc cells^36^ were injected onto the left and right flank of C57Bl/6 J Albcre; mT/mg; p53^Δ^/mice and Albcre; mT/mg; p53^Δ/Δ^ (N = 3–5/group) in 1:1 ratio of BD Matrigel and PBS. Tumors were allowed to grow till an average volume of ~40 mm^3^ following which a weekly dose of 5-FU (100 mg/kg) was administered IV for 6 weeks. Tumors were measured and volume calculated by the formula (W^2^XL)/2. Histology sections from tumors were manually counted for apoptotic and mitotic foci. Body weights of the mice were also monitored.

### Chromatin immunoprecipitation analysis (ChIP Analysis)

*In-vivo* ChIP analysis was conducted by EZ-Magna ChIP A/G (Catalog #17-10086) as per the manufacturer’s instructions. Briefly, about 50 mg of liver tissue was used and homogenized using a dounce homogenizer. The homogenized tissue was sonicated using a Misonix 3000 sonicator for approximately 9 min (on 30 sec, off 30 sec) using a microtip. The fragments were between (200–800 bp). About 1% of the chromatin was used as the input. For immunoprecipitation anti-p53 (FL393) (Cat# sc-6243 (Santa Cruz)) and IgG (Santa Cruz) were used. PCR primers were p53DBS (F-AACACTCCTTCGTTGCTCGT: R-TGAGGGACATCTGGGTTCTT) p21 (F-CCTTTCTATCAGCCCCAGAGGATACC: R-GACCCCAAAATGACAAAGTGACAA). Primers for other regions are listed in supplemental Table 1. Fold enrichment of *in vivo* ChIP was calculated by using Bio-Rad CFX96 Touch under qPCR Conditions were [95 °C for 30 sec; 55° C for 45 sec; 72°C for 45 sec] × 39 cycles. *In-vitro* ChIP analyses in HCT-116 p53^+/+^ cells were conducted according to Simple ChIP protocol according to the manufacturer’s instructions (Cell signaling Cat #9003). H3K9Ac (Cell signaling, Rabbit mAb #9649) H3K4me3 (Abcam Rabbit polyclonal #8580), H3K27me3 (Abcam Mouse mAb#6002) was used for pull-down. qPCR primers for DPYD promoter were Primer 1(F-GAAGGGAGGGAGGGAGTAGA; R-AGTGCAAGTTGTTGCTTGGA). Primer 2(F-CTCGAGTCTGCCAGTGACAA; R-CCCTAGTCTGCCTGTTTTCG) Primer 3 (F-CGAGTCGAAAACAGGCAGAC; R-TCTACTCCCTCCCTCCCTTC).

### LC/MS/MS Method for 5-Fluorouracil (5-FU) and 5-fluoro-5,6-dihydrouracil (5-FUH2)

One (1) mg/mL of stock solution was prepared in water for 5-FU, 5-FUH2 and in 60% acetonitrile (ACN) in water for 5-chlorouracil (5-CU). 10 or 100 µg/mL of intermediate stock solutions were prepared for 5-FU and 5-CU by dilution of the 1 mg/mL stock solutions with water. Intermediate combined working standard solutions (5-FU/5-FUH2) of 0.1/5, 0.2/10, 1/50, 2/100, 6/300, 10/500 in µg/mL were prepared from the stock and intermediate stock solutions in water. 2000 ng/mL of 5-CU solution was prepared in water as internal standard (IS). The mouse serum 5-FU/5-FUH2 working calibration standard were prepared by spiking 10 µL of the corresponding 5-FU/5-FUH2 intermediate working solutions (0.1/5, 0.2/10, 1/50, 2/100, 6/300, 10/500 in µg/mL) to 100 µL of blank mouse serum to generate concentrations of 5-FU/5-FUH2 at 0.01/0.5, 0.02/1.0, 0.1/5.0, 0.2/10.0, 0.6/30.0, 1.0/50.0 µg/mL. Spiking 20 µL of the 10/500 standard (5-FU/5-FUH2) to provide the calibration standard at 2.0/100.0 µg/mL. 20 µL of 5-CU (2000 ng/mL) was added to each calibration standard as internal standard. 20 µL of 5-CU (2000 ng/mL) was added to every 100 µL of mouse serum samples as an internal standard. To all the prepared working calibration standards and mouse serum samples, 10 µL of 4% phosphoric acid in aqueous, 1 mL of 16% 2-propanol in ethyl acetate were added. The glass tubes containing the mixture were vigorously mixed for 2 min on a Vortex mixer, centrifuged for 10 min at 3700 rpm/rcf, and the upper organic layers were transferred into 13 × 100 mm disposable culture tubes (Fisher). Samples were evaporated on a SpeedVac Concentrator (Savant SPD121P-115, Thermo Electron Corporation), reconstituted in 100 µL of acetonitrile, and injected into the LC/MS/MS system. 10 µL of the standards or the samples were injected on an Agilent 1100 HPLC system coupled with ABSciex 4000 Qtrap mass spectrometer. The separation was achieved with a SeQuant^TM^ ZIC^®^-cHILIC column (3.0 µm, 150 × 2.1 mm, 100 Å) at gradient of 0.0–2.0 min, 95% B; 2.0–4.0 min, 95–90% B; 4.1–6.0, 10% B and 6.1–12.0 min, 95% B with flow rate 0.3 mL/min. Mobile phase A consisted of 100 mM ammonium formate in water, and mobile phase B was ACN. The detection was conducted at electrospray negative ion mode with MRM of m/z 129.0- > 42.0 for 5-FU, m/z 131.0- > 42.0 for 5-FUH2 and m/z 145.2- > 42.0 for 5-CU.

### Cell viability assays

For cell viability assays, cells were seeded into 96-well black-walled plates at a concentration of 1–2 × 10^4^ (cancer cell lines) per well in fresh media and in a volume of 100 µL per well. At the endpoint, CellTiter-Glo™ (Promega) assays were performed according to the manufacturer’s protocol. Luminescence values were background subtracted and normalized to a No drug/No treatment control.

### Immunoblotting

The following antibodies were used: Rabbit mAb DPYD (D35A8, 1:500, Cell Signaling and Rabbit pAb Invitrogen PA5-22302), TS Rabbit mAb (D26G11, 1:1000, Cell Signaling), p53 HRP (D0-1, 1:1000, Santa Cruz Biotechnology), mouse p21 (OP-64, 1:200, Calbiochem), mouse monoclonal β-actin (A5541, 1:10000, Sigma). Secondary Goat anti-Mouse IgG (Thermo Scientific 31430, 1:10000) and Goat anti-rabbit IgG (Thermo Scientific 31460 1:10000).

### Semi-quantitative and qRT-PCR

Quantitative PCR (qPCR) for DPYD, p21 and GAPDH were conducted in triplicates on Bio-Rad CFX96 Touch for 35 cycles, PCR cycle conditions: 94 °C for 10 min, [94 °C for 30 sec; 53–55 °C for 30 sec; 72 °C for 45 sec] × 35 cycles. Gene expression values were normalized to GAPDH for each respective sample. The primer sequences used were as follows: For GAPDH, the forward primer was 5′-ACAACTTTGGTATCGTGGAAGG-3′, and the reverse primer sequence was 5′-GCCATCACGCCACAGTTTC-3′. The Human DPYD forward primer sequence was 5′-GGTGGTGATGTCGTTGGTTT-3′ and the reverse primer was 5′-GCAGAAACGGAAGCTCCATA-3′. The mouse DPYD forward primer was 5′-GACTTCAGTTTCTTCATAGTGGTGC and the reverse primer was 5′-AGCAGGGCTTTGAGTCCAGT-3′. The human p21 forward primer was 5′-CTGAGACTCTCAGGGTCGAA-3′ and the reverse primer was 5′-CGGCGTTTGGAGTGGTAGAA-3′. The mouse p21 Forward primer was 5′-TCTCAGGGCCGAAAACGGAG-3′ and the reverse primer was 5′-ACACAGAGTGAGGGCTAAGG-3′. The 18 S universal primers were purchased (Ambion #AM1716). Analysis of DPYD, p21 and 18 S in HCT-116 and HCT-116-p53^−/−^ was done by semi-quantitative PCR using the same conditions as qPCR.

### Measurement of free dUTP in cell culture in vitro

The measurement of dUTP has been previously described by Wilson *et al*. 2011^22^.

For a more detailed description of the methods see supplementary information.

### Statistical analysis

All results are presented as the mean ± SEM of data. Statistical analyses were performed by the Student’s-t-test (Two tail) and *P* values﻿ ﻿as mentioned in the figures were analyzed by Graph Pad Prism V5, unless otherwise mentioned in figure legends. Western blots are representative images of 3 independent experiments. All other experiments were conducted in triplicate unless otherwise mentioned in figure legends.

## Results

Fluorouracil (5-FU) has been a mainstay of cancer chemotherapy for over 50 years. Based on prior work implicating p53 in 5-FU induced apoptosis^19^, we hypothesized that p53 may regulate the cellular toxicity of 5-FU potentially through effects on 5-FU metabolism. In order to gain insight into how p53 could influence metabolic pathways with particular relevance for the cellular response to 5-FU, we employed an *in-silico* approach [Genomatix (http://www.Genotmatix.de/index.html)] to identify genes with potential p53 binding sites (p53BS) within and ± 20 kb upstream and downstream of the genes involved in 5-FU metabolism. We screened for p53 binding as defined by the presence of two copies of 5′-PuPuPu-CWWG-PyPyPy-3′ with or without an intervening spacer^23^. We found an enrichment of genes encoding rate-limiting enzymes in the pyrimidine catabolic pathway with p53BS. These include enzymes involved in 5-FU metabolism, whose regulation currently is poorly understood. Three genes in particular stood out, having multiple p53BS within either the 5′ promoter region or within gene introns; *DPYS*, *DPYD* and *UBP1*. We focused on DPYD as the key rate-limiting enzyme in the pyrimidine catabolic pathway.

### P53 binds to a conserved high-affinity p53BS downstream of the DPYD gene and represses gene expression

**Figure 1:**
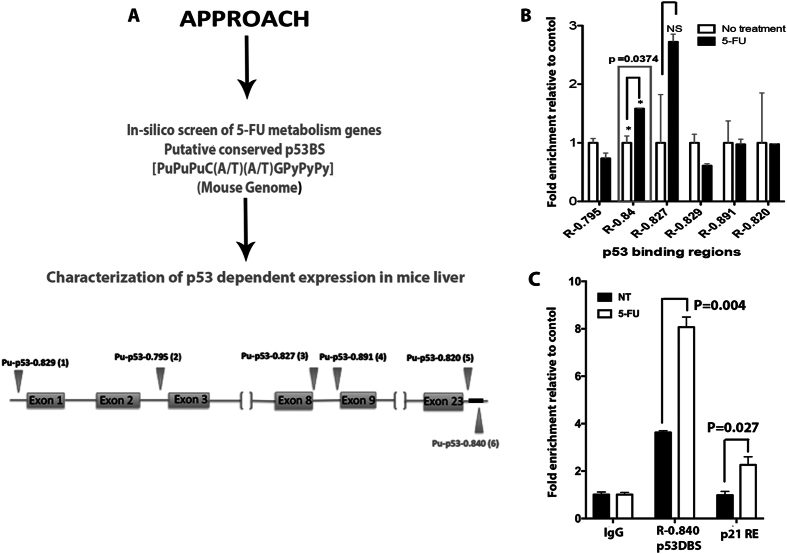
Combined *in-silico* and chromatin immunoprecipitation (ChIP) identifies p53 down-stream DNA-binding sites (p53BS) in the *DPYD* gene. (**A**) Schematic representation of the approach for *in silico* screening for putative p53BS within the *DPYD* gene and 20 kb upstream and downstream of the gene. (**B**) Fold-enrichment over untreated control of the p53BS (R-0.84) and other binding sites predicted by the *in-silico* analysis in mouse liver DNA as detected by ChIP analysis following IV 5-FU (150 mg/kg) administration for 6 hr (N = 3). (NS = Not significant) (**C**) Validation of p53DBS enrichment when treated as in (**B**), binding to p21 promoter is used as positive control. (N = 3) P-values were determined by multiple t-test.

Our *in silico* screen identified several putative half- and full-p53BS in the *DPYD* gene. However, based on the highest scoring matrix conforming to the consensus binding motif, we selected six (6) putative p53BS for further characterization **(**Fig. 1A **)**. ChIP analysis on genomic DNA isolated from the livers of mice subjected to treatment with 5-FU showed enrichment for the downstream p53BS R-0.840 (Chr1: 119451237-119451256; 5′-ACA-CATG-CTC-CAC-CATG-TTC-3′) (Fig. 1B **)**. R-0.840 was found to be conserved as a p53BS downstream (p53hDBS; chr1: 97299933–97299953; 5′-TGG-CTTG-CCT-GGG-CATG-CCT-3′) of the *DPYD* gene that was previously identified^24^. These two sequences are located at 18,319 bp (R-0.840) (p53DBS) and 15,955 bp (p53hDBS) downstream of the mouse and human *DPYD* genes respectively. In our ChIP analysis, we found that R-0.840 was enriched more than 2-fold following treatment of the mice with 5-FU (Fig. 1C). Thus it is clear that p53 increasingly occupies a p53BS downstream of the *DPYD* gene following 5-FU.

**Figure 2:**
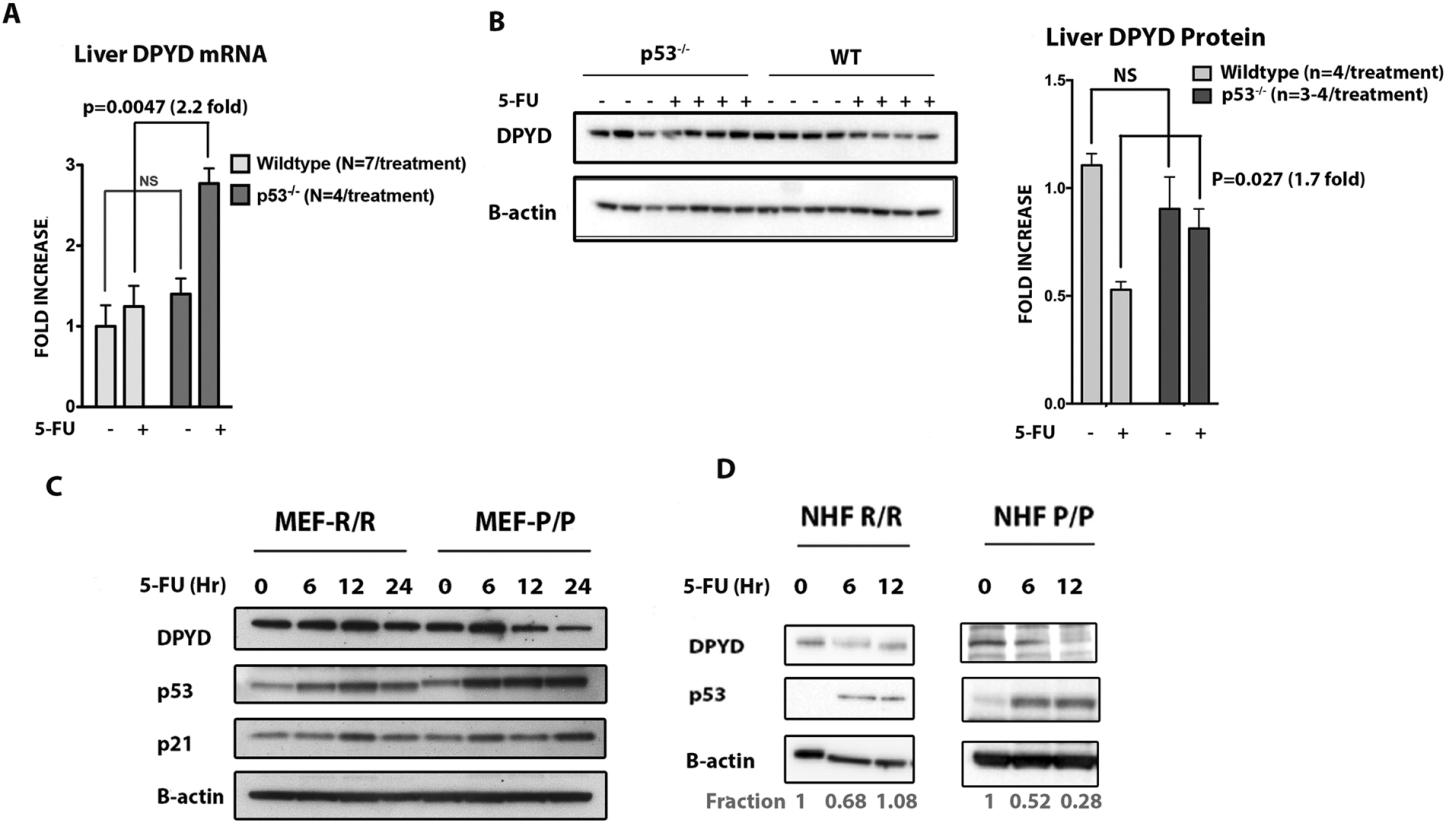
p53-dependent repression of *DPYD* expression in intact liver and impact of human p53 polymorphic variants on liver expression of *DPYD*. (**A** and **B**) Fold-change in expression of DPYD mRNA and protein in livers of p53^+/+^ (wild-type,) and p53^−/−^ mice. P-values are determined Multiple t test.and one way Anova [NT vs 5-FU in p53^+/+^ mice for DPYD mRNA is p = 0.08; DPYD protein p = 0.0021and NT vs 5-FU in p53^−/−^ mice for DPYD mRNA is p = 0.040; DPYD protein p = 0.09] (**C**) HUPKI Codon R72P MEF-R/R or MEF-P/P were treated with 5-FU (384 μM) up to 24 hr and DPYD protein expression in MEF-P/P or MEF-R/R allele was evaluated by western blot. (**D**) DPYD protein expression in Normal human fibroblast cell line harboring Codon R72P polymorphism, i.e., NHF P/P and NHF R/R is evaluated by western blot after treatment with 5-FU for the indicated times.

We have previously established a mouse model for assessing the *in vivo* response to 5-FU-based toxicities and gene expression changes in the gut^25^. Using this model, we assessed the p53-dependent expression of *DPYD* in the liver, a key organ in the biotransformation of 5-FU. Following administration of IV 5-FU, we observed that liver *DPYD* mRNA and DPYD protein expression was reduced by 2.2- and 1.7-fold respectively in *p53* ^*+/+*^ as compared to liver mRNA and protein isolated from *p53* ^−/−^ mice (Fig. 2A,B **)**. Thus, our data indicate that p53 can modulate the expression of DPYD by binding to a p53BS downstream of the *DPYD* gene.

### The R72P polymorphism in TP53 modulates DPYD expression following 5-FU exposure

We hypothesized that polymorphisms in the *TP53* gene that modulate the capacity of p53 to bind DNA could influence its ability to repress *DPYD*. Codon-72 polymorphism is a frequent polymorphism observed in the *TP53* gene and is characterized by an arginine (R) or proline (P) substitution at codon position 72. The P72 variant of p53 is capable of increased DNA binding and activation of transcription of target genes^26^. To address the impact of the R72 and P72 alleles on inhibiting the expression of *DPYD*, we used MEF’s isolated from humanized knock-in p53 mice (HUPKI) carrying variant alleles of the codon R72P polymorphism in *TP53*. Indeed, we found that the P72 allele of p53 suppresses *DPYD* expression when compared to the R72 p53 allele following 5-FU treatment in HUPKI MEF’s **(**Fig. 2C **)**. Furthermore, human fibroblasts taken from two different patients with carrying homozygous polymorphic alleles (P/P and R/R) confirmed the enhanced ability of the P72 *TP53* allele to repress *DPYD* expression following 5-FU treatment **(**Fig. 2D **)**. Taken together these results suggest that p53 inhibits *DPYD* expression and indicate that polymorphisms of the *TP53* gene could potentially indirectly alter systemic bioavailability of 5-FU that may impact the efficacy of 5-FU in cancer therapy.

### p53 represses DPYD expression following 5-FU administration in human cancer cells

**Figure 3:**
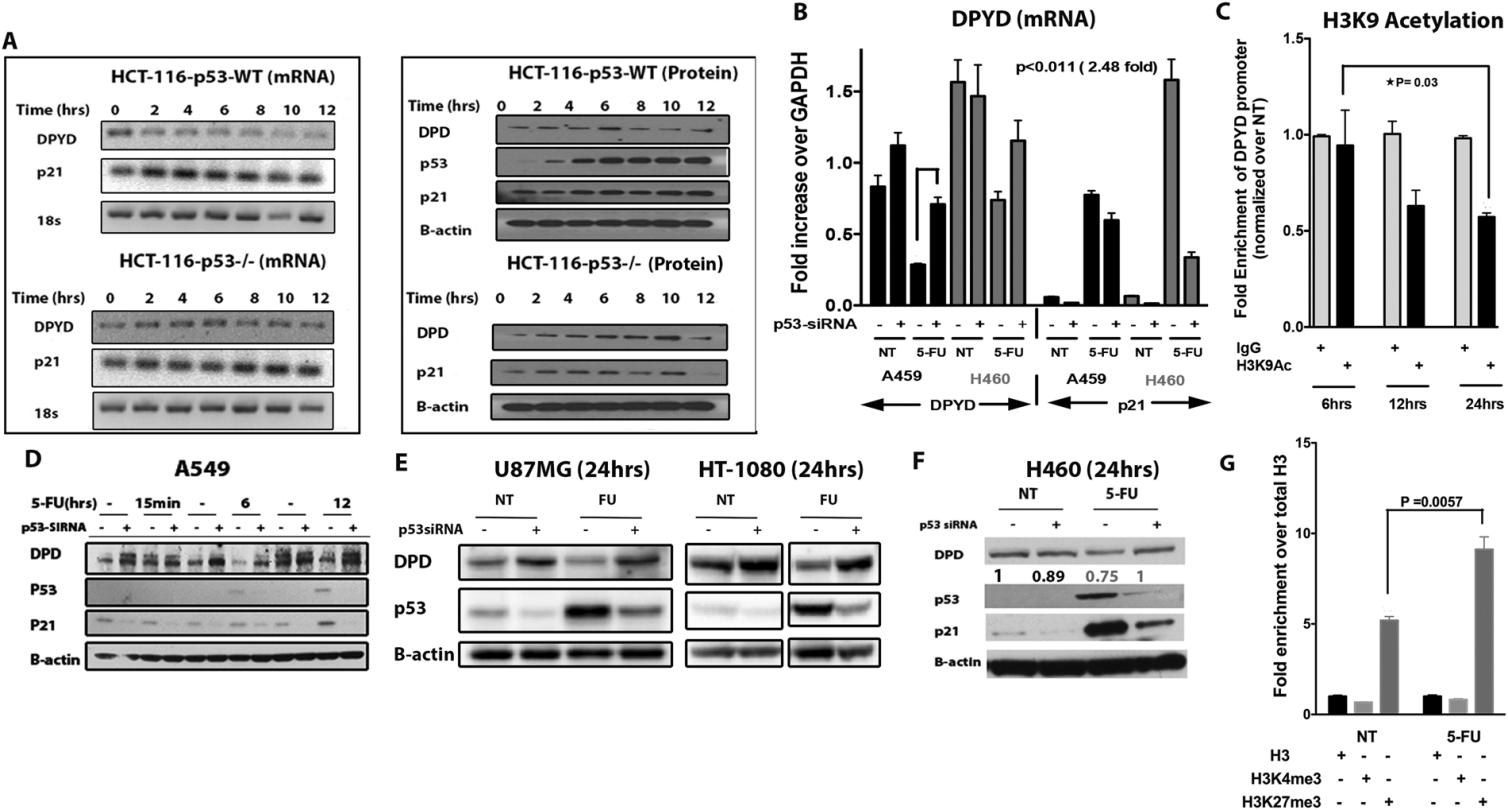
The tumor suppressor p53 represses *dihydropyrimidine dehydrogenase (DPYD)* expression. **(A)** mRNA and protein expression of *DPYD* in HCT-116 *p53* ^*+/+*^ and HCT-116 *p53* ^−/−^ cell lines at indicated times after 5-FU (384 μM) treatment. **(B)** Fold-expression of mRNA in A549 and H460 cell lines at 24 hr after 5-FU (384 μM) treatment with and without siRNA knockdown of p53 (P = 0.0011 N = 3). **(C)** H3K9 Acetylation at DPYD promoter following 5-FU treatment for indicated time points. Values are normalized in the sequence [input > IgG > total H3 > No Treatment (NT)] (N = 3). **(D**,**E**,**F)** Protein expression of DPYD in A549, U87MG, HT-1080 and H460 is shown after western blotting at the indicated time points with and without siRNA knockdown of p53. **(G)** H3K4me3 and H3K27me3 at DPYD promoter at 24hrs after 5-FU treatment. Values normalized in the sequence [input > IgG > total H3] (N = 3).

To verify that p53 mediates repression of *DPYD* expression following exposure of cells to 5-FU, we tested human cells and tissues. We first treated the isogenic colorectal cancer cell lines with functional *TP53* (HCT-116 WT) and a deleted *TP53* gene (HCT-116 p53^−/−^) with 5-FU^27^. RT-PCR analysis indicated that the mRNA expression of *DPYD* decreased over time in HCT-116 WT cells as compared to the HCT-116 p53^−/−^cells(Fig. 3A, **left panel**). This was observed at the protein level where a gradual increase in protein was noted over time following 5-FU treatment in the *TP53* ^−/−^ cells (Fig. 3A, **right panel and** Fig. S7). This was further supported by immunofluorescence staining for DPYD whose expression decreased following 5-FU exposure (Fig. S6A right panel). Since we observed increased p53 binding *in-vivo* in mouse liver to the conserved DNA-binding site, we evaluated the importance of the binding site to overall *DPYD* repression using HCT-116 WT cells. We knocked-out half of the binding site using CRISPR/Cas9 technology and analyzed for DPYD protein expression **(**Fig. S1 **)**. We found that repression of DPYD was not rescued suggesting this site alone is not sufficient to account for the observed repression. A possible reason is, since the *DPYD* gene is 850 kb in length, potentially there are other unknown p53 non-canonical binding regions that may cooperatively bring about transcriptional repression or there could be different mechanism involved inherent to tumor and normal cells. This would not at all be unexpected for a p53-regulated gene that typically harbors multiple p53 response elements scattered throughout the genomic sequence. This may include sites within promoters, several hundred base pairs or several Kb upstream of the promoters, within introns or several kb downstream of the gene. However, in order to confirm the generality of the p53-dependent *DPYD* repression after cellular exposure to 5-FU, we used the A549, H460, U87MG and HT-1080 cancer cell lines that are wild-type for *TP53* and trigger the expression of canonical p53 target genes. Indeed, the mRNA and proteins levels of *DPYD* decreased at 24 hr following treatment of the lung cancer cell lines A549 and H460 cells with 5-FU as compared to cells subjected to siRNA targeting of *TP53* (Fig. 3B and Fig. S6A,B). In a similar manner, the U87MG (glioblastoma) and HT-1080 (fibrosarcoma) cell lines also repressed *DPYD* expression in a *TP53*-dependent manner (Fig. 3D,E,F **)**. The correlative decrease of DPYD protein to its decrease in mRNA were not so apparent in the liver as it was in cancer cells, something which has previously observed^28–31^. To further investigate the transcriptional mechanism of *DPYD* repression, we evaluated H3K9 acetylation, H3K4me3 and H3K27me3 at the *DPYD* promoter region following 5-FU administration in HCT-116 and in H460 cells. H3K9 acetylation progressively decreased and was lowest at 24 hr following 5-FU treatment consistent with lower *DPYD* promoter activity **(**Fig. 3C **)**. H3K27me3 was also increased at 24 hr but no changes were observed in H3K4m3 **(**Fig. 3G and Fig. S6C). Taken together our data indicate, regardless of the genetic or epigenetic background, that p53 negatively regulates the expression of the *DPYD* gene in human cells *in vitro* and that loss of *TP53* abrogates the repression of *DPYD* expression.

### Liver specific deletion of TP53 increases systemic catabolism of 5-FU and accelerates syngeneic tumor growth

Approximately eighty (80) percent of administered 5-FU is eliminated through catabolism by hepatic DPYD^32^. In order to establish the significance of liver DPYD in limiting the bioavailability of intravenously (IV) administered 5-FU treatment we subjected mice to treatment with IV 5-FU in the presence or absence of the specific DPYD inhibitor gimeracil and monitored parameters of the acute toxic response to the drug (Fig. S2). Indeed, administration of gimeracil along with IV 5-FU triggered increased loss of body weight of mice over the course of ten (10) days as compared to IV 5-FU alone **(**Fig. S2A **)**. The group of mice subjected to the 5-FU/gimeracil combination treatment were also more moribund **(**Fig. S2B **)**. In addition, we monitored the levels of leukopenia and thrombocytopenia in mice subjected to the combination of 5-FU/gimeracil **(**Fig. S3 **)**. These data indicate that DPYD has a profound effect on the toxicity of accumulated high doses of 5-FU administered IV to mice *in vivo*.

**Figure 4:**
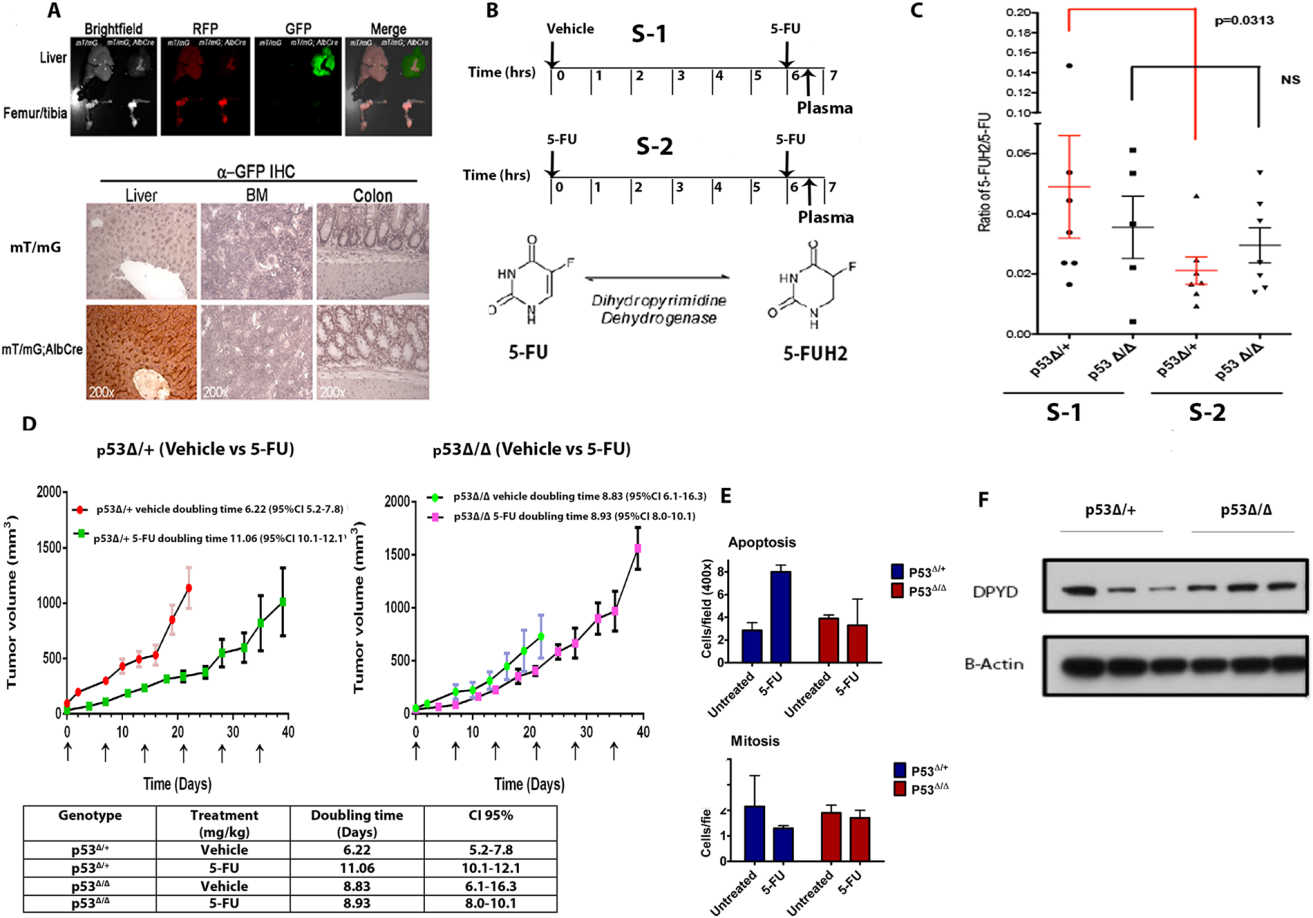
*TP53*-specific liver depletion upregulates the catabolism of 5-FU through DPYD. **(A)**. Liver specific expression of Cre in Albcre;mT/mG;p53^Δ/Δ^ mice as seen by expression of GFP and normal histology of liver, Bone marrow (BM) and colon in these mice **(B)** 5-FU treatment schedule for mice (p53^Δ/+^ and p53^Δ/Δ^) with liver specific deletion of the *TP53* gene, [S-1 = First dose Vehicle (6hrs) + second dose 5-FU(30 min)]; S-2 = [First dose 5-FU(6hrs) + second dose 5-FU(30 min)]. **(C)** Ratio of the amount of 5-FUH_2_/5-FU in plasma of liver specific *p53* ^*Δ/*^^+^ and *p53* ^*Δ/Δ*^ genotypes following the treatment plan described in (**B**) (Values represent median n = 5–7; p = 0.0313 Wilcoxon-rank-sum test). **(D)** Tumor growth delay (TGD) of syngeneic p53dmc-Ras-Myc colonocytes injected subcutaneously (s.c.) into liver specific *p53* ^*Δ/*^^+^ and *p53* ^*Δ/Δ*^ and treated with vehicle or 5-FU IV (100 mg/kg/week, for a total of 6 weeks) (N = 3–5, Doubling time calculated by exponential growth equation). **(E)** Analysis of cell death and proliferation in syngeneic tumors on *p53* ^*Δ/*^^+^ and *p53* ^*Δ/Δ*^ at the study in (**C**) as indicated by number of apoptotic and mitotic nu clei(N = 3). **(F)** Representative western blot showing expression of DPYD in liver of *p53* ^*Δ/*^^+^ and *p53* ^*Δ/Δ*^ mice with prior treatment of 5-FU for 6 hrs as indicated in (**B**) and (**C**).

We further explored if the repression of *DPYD* expression by p53 *in vivo* could modulate the pharmacokinetics of 5-FU. We hypothesized that targeting of *DPYD*-expression through deletion of *TP53* in hepatocytes *in vivo* may impact the systemic bioavailability of 5-FU. To this end we generated mice with liver-specific deletion of the *TP53* gene i.e., through the use of mice expressing *Cre* recombinase under the control of the Albumin promoter^33^. Indeed, Albcre;mT/mg;p53^Δ/Δ^ mice showed expression of *Cre* recombinase in hepatocytes and no histological changes were detected in other organs following deletion of *TP53* in the liver (Fig. 4A **)**. The plasma half-life of 5-FU is approximately ~20 min^34, 35^. Therefore, in order to measure the impact of *DPYD* repression on 5-FU bioavailability, we followed a treatment schedule in which mice were either given Vehicle or 5-FU for the first 6 hrs, i.e. a time point at which DPYD expression is repressed (Fig. 2B), followed by second dose of 5-FU for 30 min. As expected, a lower ratio of 5-FUH_2_/5-FU was observed in Albcre;mT/mg;p53^Δ/+^ mice as compared to the Albcre;mT/mG;p53^Δ/Δ^ mice **(**Fig. 4B,C **)**. Western blot analysis revealed expression levels of DPYD in these mice consistent with our earlier observation of repression of *DPYD* by p53 (Fig. 4F). We further investigated whether the apparent decrease in 5-FU catabolism indicated by a lower plasma ratio of 5-FUH_2_/5-FU in the presence of wild-type *TP53* could have a functional consequence on the *in vivo* therapeutic response to 5-FU. We used syngeneic (C57BL6/J) malignantly transformed mouse colonocytes to model colorectal cancer with mutated KRAS, p53 and Myc-overexpression (p53dmc-Ras-Myc)^36^. p53dmc-Ras-Myc cells were injected subcutaneously in the flanks of mice and the mice were subjected to treatment with 5-FU (100 mg/kg/week for 6 weeks) with a follow-up time of up to 40 days. Indeed, Albcre;mT/mg;p53^Δ/+^ mice showed a significant increase in the tumor doubling-time (vehicle versus 5-FU-treated tumor doubling-times were 6.22 versus 11.06 days, respectively) following treatment with 5-FU **(**Fig. 4D **)**. In comparison Albcre;mT/mg;p53^Δ/Δ^ mice displayed a clearly blunted response to 5-FU when compared to vehicle-treated controls (vehicle compared to 5-FU-treated tumor doubling-times were 8.83 vs. 8.93 days, respectively). Furthermore, tumors from 5-FU-treated Albcre;mT/mg;p53^Δ/+^ mice exhibited increased levels of apoptosis and decreased levels of mitosis when compared to tumors from 5-FU-treated Albcre;mT/mg;p53^Δ/Δ^ mice **(**Fig. 4E **)**. To eliminate the possibility of hepatotoxicity affecting the outcomes we analyzed liver sections from both 5-FU-treated and -untreated mice and found no differences in tissue histology, or any change in body weight (Fig. S4). Taken together these results strongly implicate p53 in controlling liver catabolism and therapeutic efficacy of 5-FU.

### Repression of DPYD by p53 is a consequence of thymidylate synthase inhibition and thymidine deficiency

**Figure 5:**
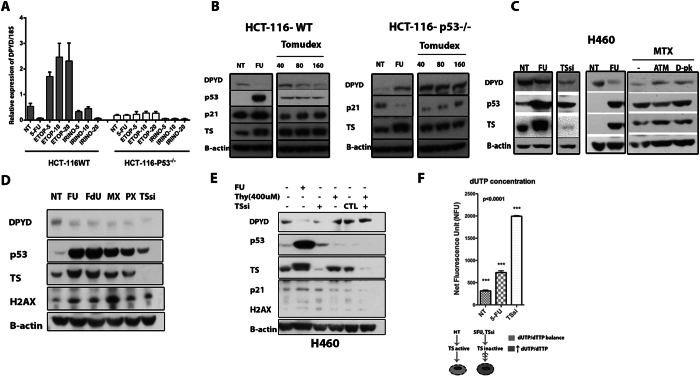
p53 represses the expression of *DPYD* specifically following thymidylate synthase (TS) inhibition due to thymidine deficiency. (**A**) mRNA expression of DPYD following Etoposide and CTP-11 in HCT-116 *p53* ^*+/+*^ and *p53* ^−/−^ colorectal cancer cells (N = 3). (**B**) DPYD Protein expression following TS inhibition in HCT-116 *p53* ^*+/+*^ and *p53* ^−/−^ cell lines treated with 5-FU and Tomudex (Raltitrexed) for 24 hr. (All lysates were evaluated on same gel). (**C**) Rescue of *DPYD* protein repression by inhibition of the DNA damage response, with ATM (KU-55933) and DNA-PK (D-PK) (NU7026) inhibitors for 24 hr of H460 cells treated with methotrexate (MTX). (**D**) Repression of *DPYD* protein expression with various TS inhibitors [Fluorouracil (FU), Fluorodeoxyuridine (FdU), methotrexate (MTX), Pemetrexed (PX),] and TS knockdown (TSsi). (**E**) Rescue of *DPYD* protein repression by addition of thymidine-H460 cells that were incubated with thymidine following treatment with 5-FU or TS knockdown for 24 hrs. (**F**) Increase in dUTP levels following treatment with 5-FU and TSsi.

To better our understanding of the upstream signaling events behind the p53-dependent repression of *DPYD* by p53 we asked whether this was specifically related to the activity of 5-FU or just simply part of the cellular DNA damage response (DDR). Treating HCT-116 cells with different chemotherapeutic agents indicated that the repression of *DPYD* was not a result of DNA damage *per se* since treatments with the Topoisomerase-I and -II poisons irinotecan (CPT-11) and etoposide respectively did not reveal a p53-dependent inhibition of *DPYD* expression **(**Fig. 5A **)**. Both irinotecan and etoposide caused induction of *DPYD* expression in a somewhat p53-dependent manner. The therapeutic effect of 5-FU is considered to arise from incorporation of 5-FU in DNA, RNA and mainly by inhibition of thymidylate synthase (TS)^15^. We hypothesized that the effect of 5-FU to repress *DPYD* may stem from TS targeting and the resulting depletion of thymidine pools that may trigger p53 activation. To address this specifically, we treated HCT-116-WT and HCT116 *TP53* ^−/−^ cells with 5-FU and Tomudex (Raltitrexed), a specific inhibitor of TS. 5-FU forms an inactive ternary complex with TS, which causes a slight upward shift in the TS band signifying TS inhibition^37^, whereas TS inhibitors such as Tomudex, Pemetrexed (PX; Alimta®) and methotrexate relieve feed-back inhibition of TS mRNA translation causing modest increases in TS protein expression^37–40^. We found a p53- and dose-dependent repression of *DPYD* expression following 5-FU and Tomudex-treatment **(**Fig. 5B **)**. As an alternative approach to block TS, we employed the TS-inhibitor methotrexate, not a direct inhibitor *per se* and direct siRNA targeting TS. Treatment of H460 cells with methotrexate and with TS siRNA generated similar results of p53-dependent *DPYD* repression as seen with 5-FU and Tomudex (Fig. 5C **)**. The results were further expanded to Pemetrexed (PX; Alimta®) and Fluorodeoxyuridine (FdU; floxuridine) that also selectively act on TS (Fig. 5D **)**. As data using five (5) different inhibitors yielded similar results with respect to p53-dependent inhibition of *DPYD* expression, we sought to determine whether thymidine supplementation could overcome the p53-mediated repression of *DPYD*. As expected addition of exogenous thymidine restored DPYD protein expression following targeting of TS with siRNA **(**Fig. 5E **)**. As a control experiment to directly verify the impact of siRNA-mediated targeting of TS we analyzed changes in intracellular nucleotide pools. Inhibition of TS is known to cause reduced conversion of dUMP to dTMP and as a result compensatory increased conversion of dUMP to dUTP^15^. We found the levels of free dUTP increase in cells subjected to siRNA-targeting of TS and 5-FU **(**Fig. 5F **)**. Interestingly, targeting of TS by siRNA was approximately three (3) times more effective than 5-FU to generate increased levels of cellular dUTP, indicating the functional relevance of the TS-inhibition approach. Taken together the data suggest that following efficient TS-inhibition, p53 may specifically repress the expression of DPYD protein and downregulate pyrimidine catabolism.

### p53-dependent repression of DPYD is dependent on ATM and DNA-PK signaling following TS inhibition

**Figure 6:**
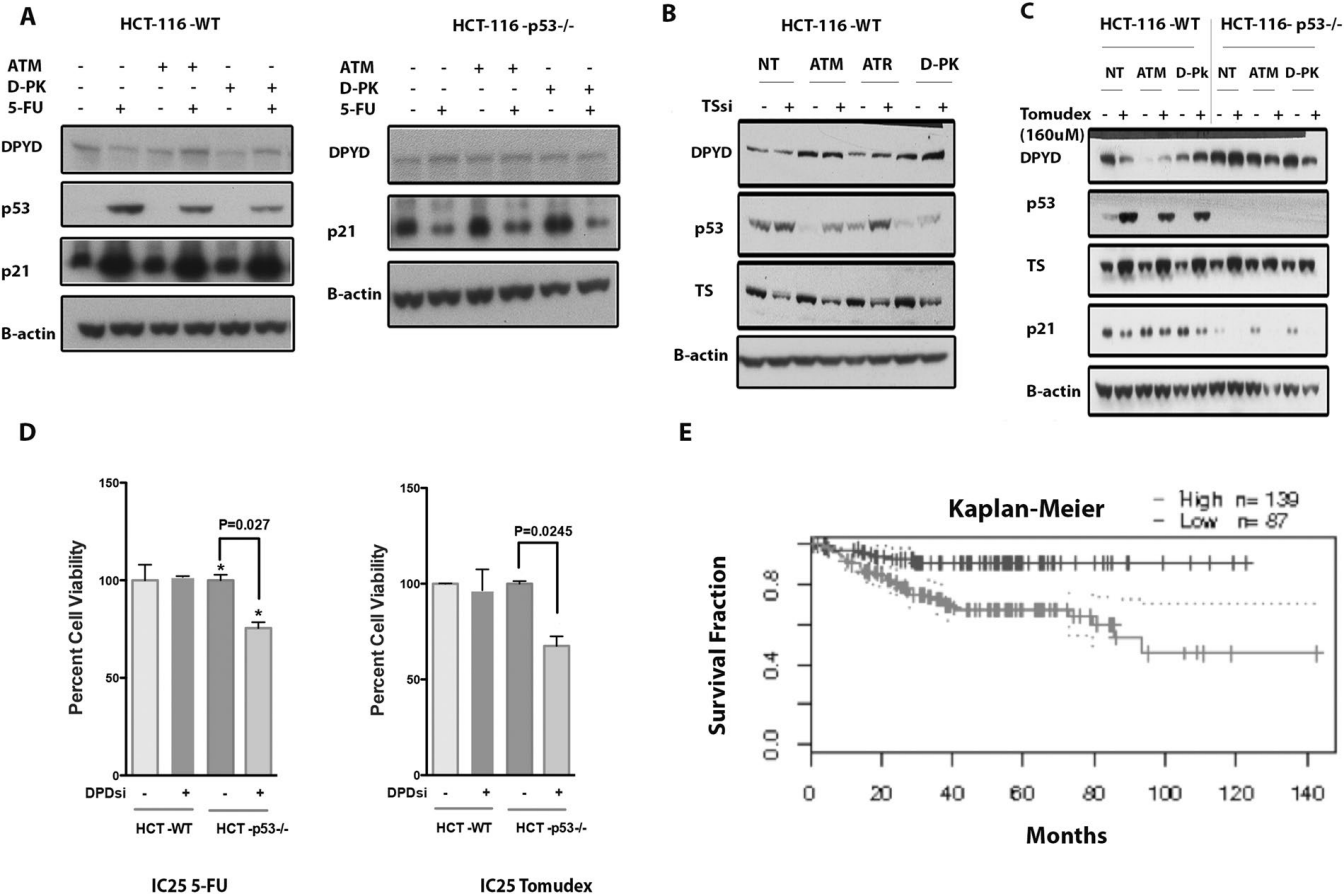
p53-dependent repression of *DPYD* is dependent on signaling from ATM and DNA-PK following TS inhibition. **(A)** Rescue of *DPYD* repression by inhibition of DNA damage response, HCT-116-WT and *p53* ^−/−^ cells treated with 5-FU, with ATM (KU-55933) and DNA-PK (NU7026) inhibitors for 24 hrs. **(B**,**C)** Co-treatment with ATM and DNA-PK inhibitors relieves inhibited DPYD expression following targeting of TS with siRNA (TSsi) and Tomudex. The effects of siRNA targeting of *DPYD* on 72-hr cell-viability of HCT-116-WT and *p53* ^−/−^ cells following treatment with isotoxic doses of 5-FU (**D**) and Tomudex. **(E)** High expression of *DPYD* mRNA predicts poor disease-free survival in colorectal cancer patients mainly with Duke Stage B and C as analyzed from the GSE14333 (Melbourne) data set. P-value was calculated by cox regression analysis (Cox P-Value = 0.00105).

A decrease in the thymidine pools can result in nucleotide imbalance and cause DNA damage that in turn may lead to p53 activation^41^. We hypothesized that blocking upstream DDR kinases that impact on p53 stabilization potentially could relieve inhibition of *DPYD* repression. We treated HCT-116 *TP53* WT or *TP53* ^−/−^ cells with inhibitors of ATM (KU-55933) or DNA-PK (NU7026) in the context of 5-FU-induced damage. We observed that in wild-type *p53*-expressing cells the 5-FU induced repression of *DPYD* was alleviated when cells were co-treated with the kinase inhibitors (Fig. 6A). By contrast, in *TP53* ^−/−^ cells no change in the expression of *DPYD* was observed following co-treatment with KU-55933 or NU7026. We extended our analysis to include siRNA to TS (TSsi) and Tomudex, **(**Fig. 6B,C **)**. The results indicate that following more selective targeting of TS, p53-dependent inhibition of *DPYD* expression requires functional DNA-PK and ATM. Despite some observed discrepancies between DNA damaging chemotherapeutics such as etoposide, CPT-11 and 5-FU in the triggering p53-dependent repression of *DPYD* (Fig. 5F), our data indicate that key DNA-damage signaling kinases such as ATM and DNA-PK are required to signal p53-dependent repression of *DPYD* expression following TS inhibition. In order to functionally validate how DPYD influences the cancer cell intrinsic response to TS inhibitory drugs we targeted *DPYD* expression in *p53*-null cancer cells with siRNA and subjected them to treatment with Tomudex and 5-FU. Knockdown of *DPYD* in HCT-116-*p53* ^−/−^ cells significantly sensitized them to the toxic effects of 5-FU and Tomudex **(**Fig. 6D **)**. Thus, it appears that elevated levels of DPYD may contribute to a resistance phenotype to TS inhibitors observed in cancers that have lost functional p53. Furthermore, this may indicate that *DPYD* can confer drug resistance to TS inhibitory drugs that is independent of systemic catabolism and reduced bioavailability.

### DPYD mRNA expression correlates with poor disease free survival in colorectal cancer

We analyzed whether tumor DPYD mRNA expression could predict poor outcome in colorectal cancer patients treated with chemotherapy. Analysis of the GSE14333 data-set^42^ in 226 colorectal cancer patients comprised mainly of Duke Stage B and C cancers revealed that high *DPYD* mRNA expression correlates with poor disease free survival as compared to those expressing low *DPYD* mRNA levels **(**Fig. 6E and Supplemental Table 2 **)**. Analysis of a TCGA cohort for correlation between *TP53* and *DPYD* expression revealed a strong inverse trend, where higher *TP53* expression showed lower *DPYD* expression along with poorer survival rate in patients having *TP53* ^Low^/*DPYD* ^High^ vs *TP53* ^High^/*DPYD* ^Low^ **(**Fig. S5 **)**. However, these analyses did not reach statistical significance.

**Figure 7:**
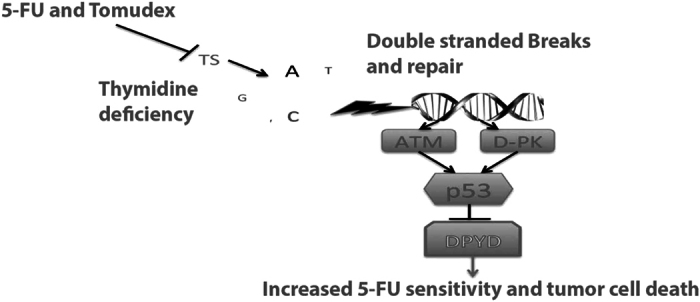
Model depicting the repression of DPYD by p53 following thymidylate synthase (TS) inhibition, dTTP imbalance and DNA damage. P53 transcriptionally represses DPYD expression and negatively impacts the catabolism of pyrimidines following inhibition of TS. In extension, this results in an imbalance in the dTTP/dUTP ratio and a need to salvage cellular levels of dTTP to maintain uninterrupted DNA replication and repair. However, in the presence of 5-FU this might cause increased cell death due to reduced catabolism and increased incorporation of the 5-FU metabolites FdUTP and FUTP into DNA and RNA respectively.

Taken together, our data suggests that an imbalance in the cellular nucleotide pool resulting from reduced levels of thymidine required for the synthesis of DNA triggers p53-dependent inhibition of the key rate-limiting enzyme of pyrimidine catabolism DPYD which in turn reduces 5-FU catabolism (Fig. 7).

## Discussion

We show for the first time that the p53 tumor suppressor protein controls 5-FU catabolism by repressing the expression of the key rate limiting enzyme in pyrimidine degradation DPYD. p53 has a well-documented function in the cell death response following 5-FU treatment in pre-clinical experiments *in vitro* and *in vivo* ^19, 43^. In clinical settings *TP53* mutation status has been correlated with survival following 5-FU-based chemotherapy^17–19^. However, it has been difficult to identify key genes downstream of p53 that predict the 5-FU response in patients *in vivo* and *TP53* mutation status may not necessarily be a predictor of the response to other chemotherapy agents. This may partly be due to the complexity of the mechanism of action of 5-FU that ranges from interference with both DNA and RNA synthesis through direct incorporation in such polynucleotide strands and subsequent repair thereof and inhibition of dTTP production through targeting of TS^44^.

A critical determinant of the efficacy of 5-FU treatment directly relates to the expression of key metabolic genes that are responsible for the biotransformation of the drug. DPYD is an important catabolic enzyme that limits the bioavailability of 5-FU to therapeutically relevant anabolic pathways and has been linked to 5-FU efficacy and toxicity^45^. We show that high expression levels of DPYD is linked to poor outcomes in colorectal cancer. Interestingly, the link we have uncovered between *TP53* and *DPYD* suggests that expression of both of these genes may serve as markers in determining the response to 5-FU. Furthermore, observations in humanized *TP53* knock-in mice and patient samples suggest that polymorphisms in the *TP53* gene can influence the inhibition of *DPYD* expression. Based on our data, the capability of the *P72 TP53* allele, which is a more active transcriptional variant of *TP53*, in repressing *DPYD* expression over the *R72 TP53* allele suggest that p53 may alter the 5-FU drug sensitivity heterogeneously in the general population through its impact on 5-FU catabolism of normal tissues such as the liver (Fig. 2C,D). The tumor response observed in our syngeneic mouse model clearly demonstrates the importance of systemic effects of this interaction (Fig. 4D). However, it should be noted that the syngeneic tumors in our study expressed low levels of DPYD in comparison to liver tissue (data not shown). Evidence suggests that some tumors express high levels of DPYD compared to surrounding normal tissues as something that may in particular be true for colorectal cancers that metastasize to the liver^46^. Overexpression of *TS* and *DPYD* is seen frequently in colorectal tumors^47, 48^. It is generally known in the clinical setting that tumors with low *DPYD* expression, low *TS* expression and that are wild-type for *TP53* show a favorable response rate following treatment with 5-FU^49^. Subsequently, loss of functional p53-signaling in colorectal cancer, a typical late-stage event in the disease, may fail to suppress *DPYD* expression and add another level of complexity to 5-FU treatment by contributing to drug resistance. This idea is supported by the *DPYD* gene expression profile in p53 WT cell lines **(**Fig. 3 **)** as well as in advanced stage colorectal tumor patients where higher expression of *DPYD* predicts poor disease-free survival **(**Fig. 6E and Fig. S5 **)**. It remains to be seen to what extent tumor levels of DPYD can make a significant contribution to 5-FU catabolism *in vivo*.

Our data in tumor cell lines suggest that inhibition of TS by 5-FU, methotrexate, Tomudex or Pemetrexed causes p53-dependent repression of *DPYD* expression (Fig. 5D). We provide evidence that repression by p53 of *DPYD* expression is related to changes in the dUTP/dTTP ratio and signaling from ATM and DNA-PK following TS-inhibition unlike other DNA damaging agents. A potential compensatory response to the reduction in dTTP levels, as a result of TS-inhibition, could be to reduce catabolism of pyrimidines to salvage thymine and uracil necessary for dTTP synthesis and DNA replication recovery. Suppression of pyrimidine catabolism by p53 following nucleotide imbalance may be another way for the *TP53* tumor suppressor to control the integrity of DNA synthesis by favoring nucleotide salvage of thymidine and prevent errors during replication. However, since blocking pyrimidine catabolism would also affect catabolism of 5-FU, this would in turn cause an increase in 5-FU bioavailability emphasizing the deleterious effects in wild-type *p53*-expressing cells. In line with this, siRNA targeting of *DPYD* in *TP53*-null cells sensitizes to chemotherapeutics that target TS such as Tomudex (Fig. 6D).

Our data suggest that inhibiting DPYD would work synergistically with TS inhibitors. In support of our observations, use of a clinical DPYD inhibitor gimeracil as a component of the oral S-1 (tegafur, gimeracil and oteracil) mix has already proved promising in many solid tumors and is approved in more than 50 countries but not in the US. Early indications of synergy between S-1 and TS inhibitors have been reported for 5-FU resistant tumors^50^. S-1 has been combined with HDAC inhibitors, which are known to downregulate TS expression^51^. Moreover, targeting DPYD with the oral irreversible inhibitor eniluracil, has significantly improved PFS and OS of patients with metastatic breast cancer in comparison to patients who were refractory to capecitabine (oral 5-FU)^12^. Eniluracil also limited the frequency of hand-foot syndrome, a toxicity phenotype believed to result from metabolites of 5-FU catabolism. Considering that recent evidence indicates that DPYD may play a role in breast cancer metastasis^2^, it would be interesting to determine if a DPYD inhibitor might provide added benefit to patients by limiting toxicity as well as targeting tumors that undergo EMT.

In conclusion, our study provides the first evidence for a role of the tumor suppressor p53 protein in downregulating pyrimidine and 5-FU catabolism by repressing *DPYD* gene expression following TS inhibition. These effects are not observed with other DNA damaging chemotherapeutic drugs like the topoisomerase inhibitors etoposide or irinotecan. Further studies would need to evaluate the interplay between combined use of 5-FU and irinotecan as compared to 5-FU alone with regard to p53-dependent regulation of *DPYD*. The idea that mutant p53 could through derepression up-regulate *DPYD* as a resistance mechanism to 5-FU treatment is also a focus of future research as 5-FU is used in multiple consecutive regimens in the therapy of evolving colorectal tumors. Overall our data document a role for tumor suppressor p53 in controlling pyrimidine catabolism through *DPYD*, particularly following metabolic stress imposed by nucleotide imbalance, and signaling effects through DNA-PK and ATM. The findings have implications for the toxicity and efficacy of the cancer therapeutic 5-FU. Previous findings from our lab have demonstrated that monitoring 5-FU levels can minimize toxicity and improve outcomes^52^ and thus this study can provide some avenues for future design of potentially more effective treatments or treatment monitoring.

## Electronic supplementary material

Supplementary Information
