# Author Correction: P53 represses pyrimidine catabolic gene *dihydropyrimidine dehydrogenase* (*DPYD*) expression in response to thymidylate synthase (TS) targeting

**DOI:** 10.1038/s41598-025-16375-w

**Published:** 2025-08-26

**Authors:** Prashanth Gokare, Niklas K. Finnberg, Phillip H. Abbosh, Jenny Dai, Maureen E. Murphy, Wafik S. El-Deiry

**Affiliations:** 1 Laboratory of Translational Oncology and Experimental Cancer Therapeutics, Department of Hematology/Oncology and Molecular Therapeutics Program, Fox Chase Cancer Center, Philadelphia, PA 19111 USA; 2 Penn State Hershey Cancer Institute, Penn State Hershey Medical Center, 500 University Dr, Hershey, PA 17033 USA; 3 Molecular and Cellular Oncogenesis Program, The Wistar Institute, Philadelphia, PA 19104 USA

Correction to: *Scientific Reports* 10.1038/s41598-017-09859-x, published online 29 August 2017

There are errors in this Article. Due to an error during figure assembly, in Figure 3A the beta-actin blot for “HCT-116-p53-/- (Protein)” is showing a higher exposure of the beta-actin blot of “HCT-116-p53-WT (Protein)”.

The beta-actin blot for “HCT-116-p53-/- (Protein)” has been replaced and the corrected Figure 3 is shown below.

**Figue 3:**
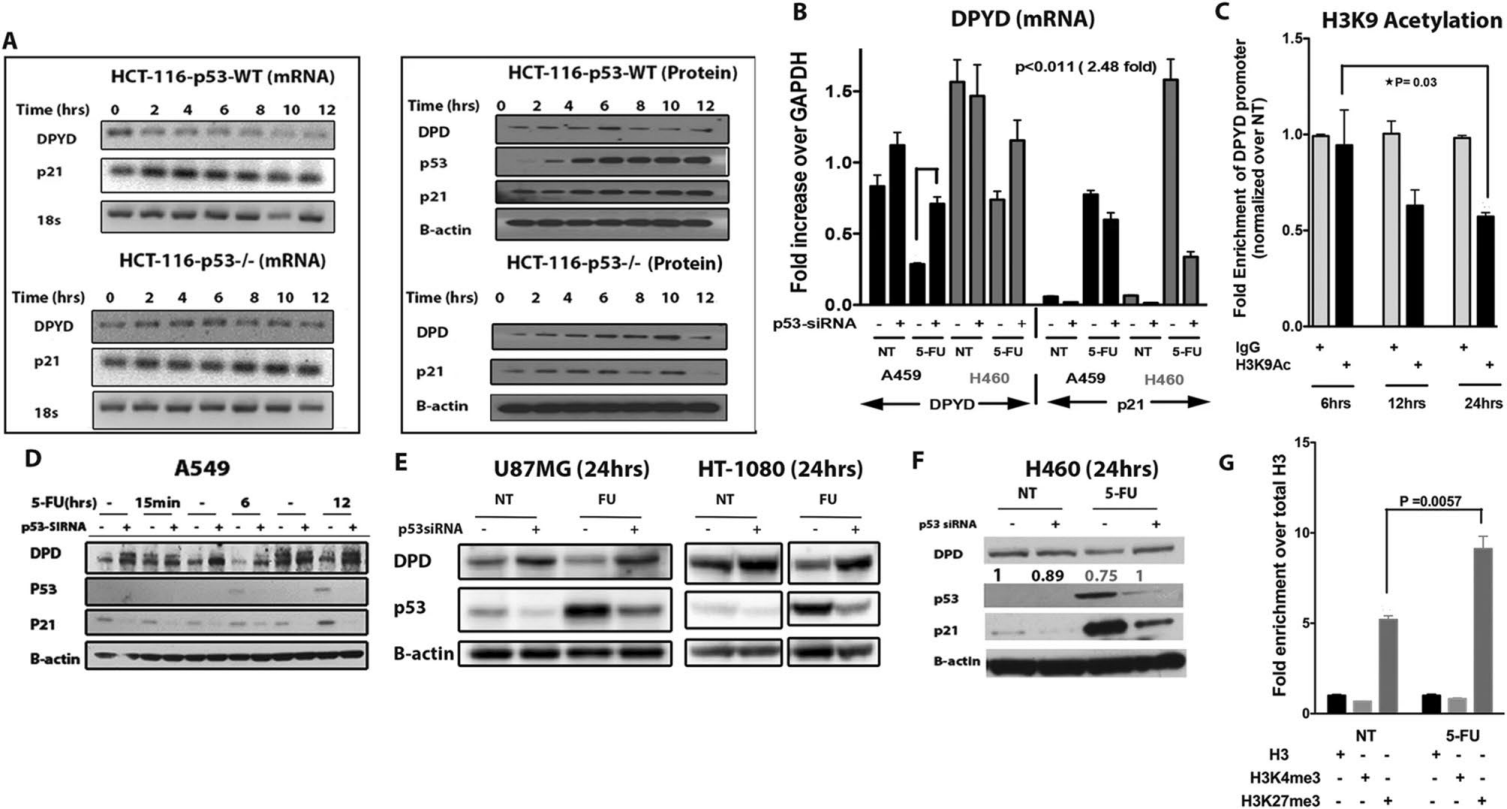
The tumor suppressor p53 represses *dihydropyrimidine dehydrogenase (DPYD)* expression. **(A)** mRNA and protein expression of *DPYD* in HCT-116 *p53* ^+*/*+^ and HCT-116 *p53* ^−/−^ cell lines at indicated times after 5-FU (384 μM) treatment. **(B)** Fold-expression of mRNA in A549 and H460 cell lines at 24 h after 5-FU (384 μM) treatment with and without siRNA knockdown of p53 (P = 0.0011 N = 3). **(C)** H3K9 Acetylation at DPYD promoter following 5-FU treatment for indicated time points. Values are normalized in the sequence [input > IgG > total H3 > No Treatment (NT)] (N = 3). **(D**,**E**,**F)** Protein expression of DPYD in A549, U87MG, HT-1080 and H460 is shown after western blotting at the indicated time points with and without siRNA knockdown of p53. **(G)** H3K4me3 and H3K27me3 at DPYD promoter at 24h after 5-FU treatment. Values normalized in the sequence [input > IgG > total H3] (N = 3).

Additionally, in Figure 5C the TS blot in the third column of blots is mistakenly duplicated from the p53 blot. The correct TS blot was already included in Supplementary Figure 8G, but was erroneously labelled as belonging to Figure 5B.

The corrected Figure 5 is shown below.

**Figure 5:**
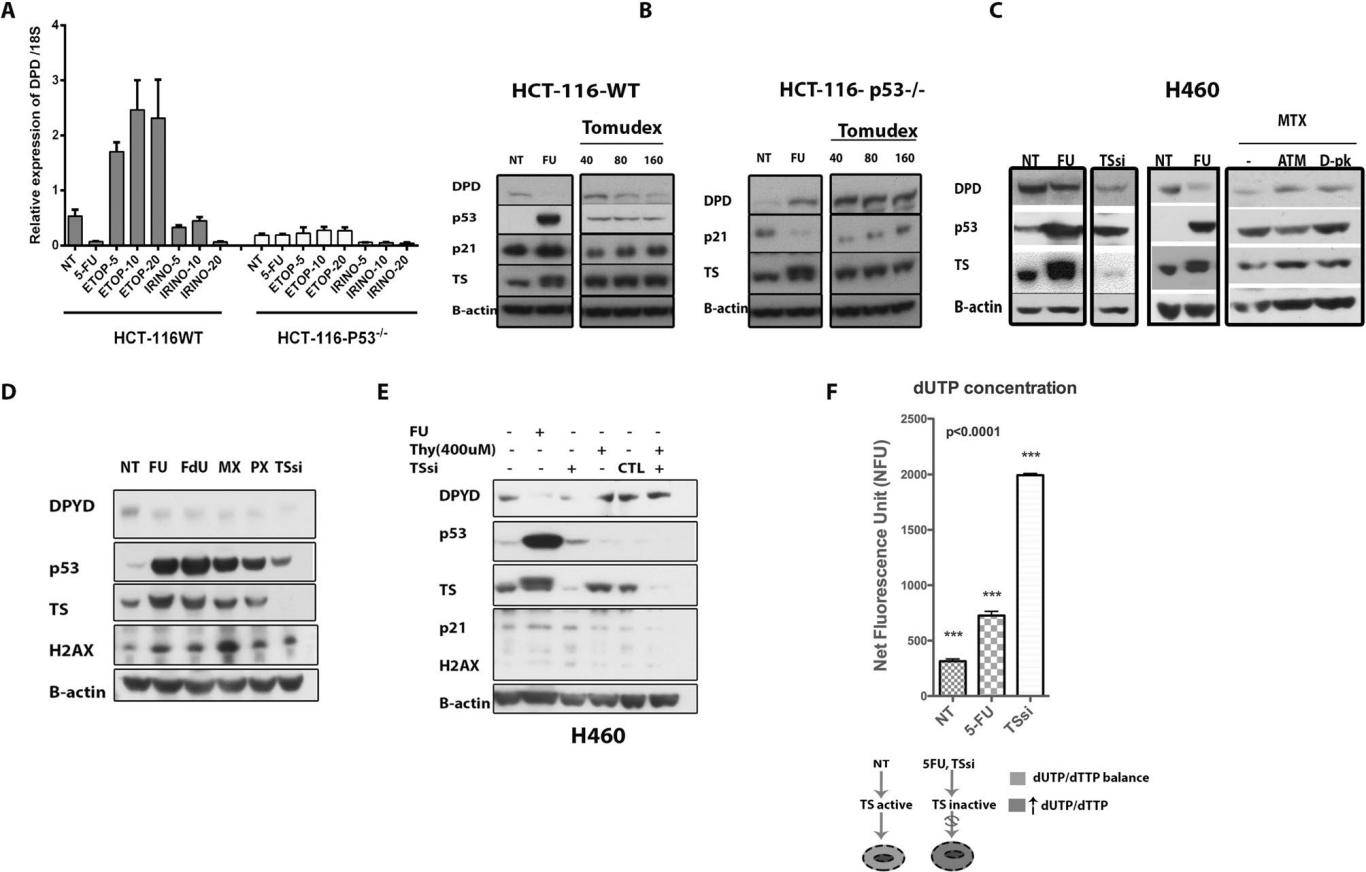
Original western blotting images. The figures in the main text corresponding to blots are indicated on top of the blot.

The corresponding underlying data for the beta-actin blot in Figure 3A has been replaced in supplementary Figure 8D. Supplementary Figure 8G and H have been amended to indicate the Figures panels they belong to. The corrected Supplementary Figure 8 is shown below.

**Figure S8:**
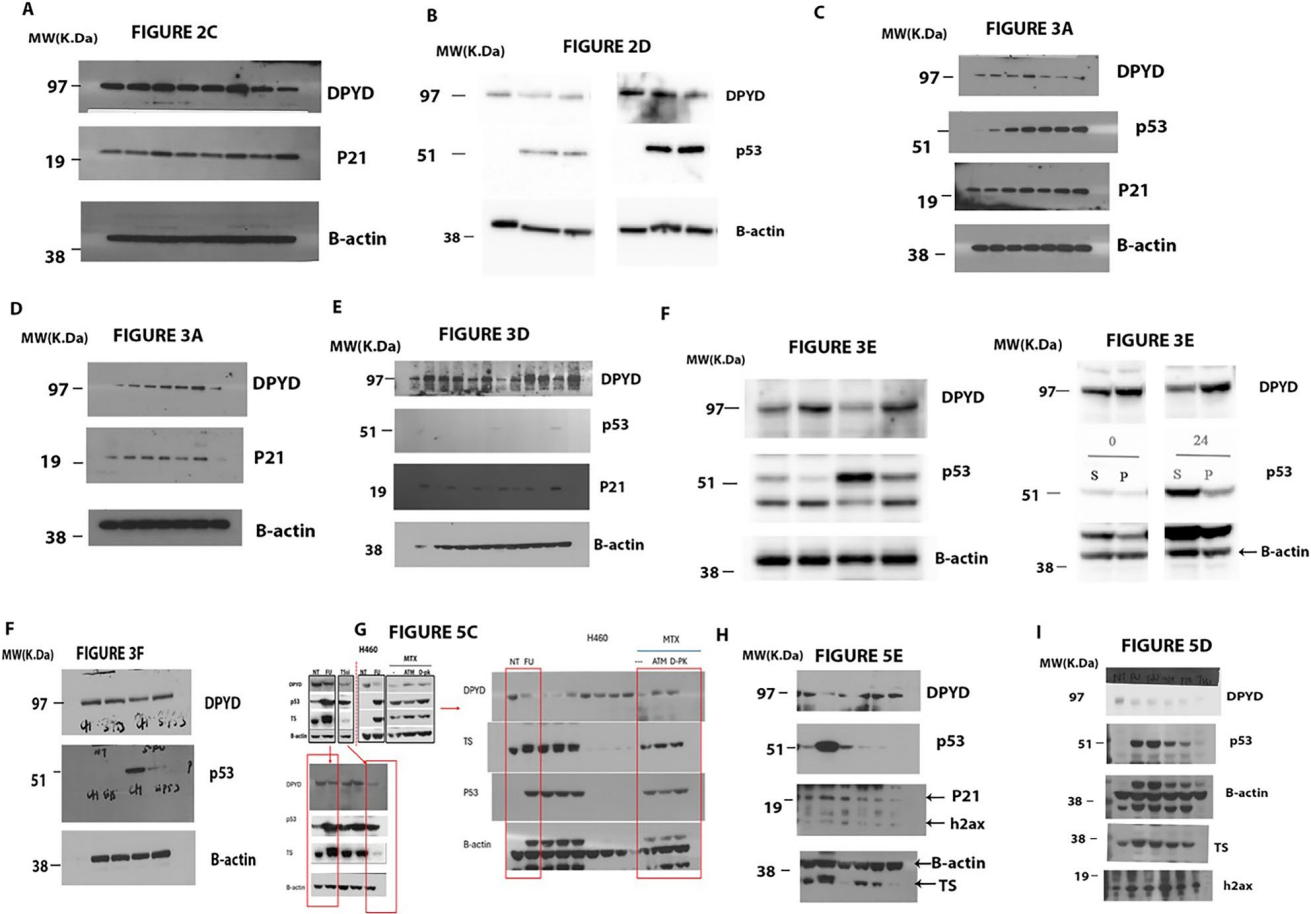
p53 represses the expression of *DPYD* specifically following thymidylate synthase (TS) inhibition due to thymidine deficiency. (**A**) mRNA expression of DPYD following Etoposide and CTP-11 in HCT-116 *p53* ^+*/*+^ and *p53* ^−/−^ colorectal cancer cells (N = 3). (**B**) DPYD Protein expression following TS inhibition in HCT-116 *p53* ^+*/*+^ and *p53* ^−/−^ cell lines treated with 5-FU and Tomudex (Raltitrexed) for 24 h. (All lysates were evaluated on same gel). (**C**) Rescue of *DPYD* protein repression by inhibition of the DNA damage response, with ATM (KU-55933) and DNA-PK (D-PK) (NU7026) inhibitors for 24 h of H460 cells treated with methotrexate (MTX). (**D**) Repression of *DPYD* protein expression with various TS inhibitors [Fluorouracil (FU), Fluorodeoxyuridine (FdU), methotrexate (MTX), Pemetrexed (PX),] and TS knockdown (TSsi). (**E**) Rescue of *DPYD* protein repression by addition of thymidine-H460 cells that were incubated with thymidine following treatment with 5-FU or TS knockdown for 24 h. (**F**) Increase in dUTP levels following treatment with 5-FU and TSsi.

