# Supplementary material for: P53 represses pyrimidine catabolic gene dihydropyrimidine dehydrogenase (DPYD) expression in response to thymidylate synthase (TS) targeting
Source: Sci Rep. 2017 Aug 29;7:9711. doi: 10.1038/s41598-017-09859-x (PMC5575263; doi:10.1038/s41598-017-09859-x)
Supplement: Supplementary file 1 — Supplementary Information [file 41598_2017_9859_MOESM1_ESM.pdf]

P53 represses pyrimidine catabolic gene *dihydropyrimidine dehydrogenase* (*DPYD*) expression following thymidylate synthase (TS) targeting.

Prashanth Gokare, Niklas K. Finnberg, Phillip H. Abbosh, Jenny Dai, Maureen E. Murphy and Wafik S. El-Deiry

Supplementary Information

MATERIAL AND METHODS

Knockout of the p53 binding site using CRISPR:

We designed and tested 3 sgRNA sequences for the p53 binding site and selected the best one i.e sg1 ATACAACCTATGGCTTGCCT, which was cloned into pLentiCRISPR-E (Addgene Plasmid #78852). The lentivirus was generated in HEK-293T cells in 10cm dish. Viral supernatant was added to HCT-116WT cells in 6well plates (Ratio 1:1) and then selected with puromycin at (1ug/ml) for 15 days to get pooled clones of p53BSKO cells.

H&E Staining: Blinded analysis of liver sections from two 5-FU treated and Vehicle treated mice was performed by the pathologist.

Survival Curve Analysis

Two subsets of TCGA colorectal patients were defined, based on levels of RNA expression (RSEM-normalized expression from RNA-seq data for COAD and READ

datasets). Groups of interests were those patients with (1) high TP53 and low DPYD expression (relative to the overall medians for each gene), and (2) low TP53 and high DPYD expression. Overall survival was compared for these two groups using Kaplan-Meier analysis, and the calculated p-value is from a log-rank test.

Scatterplot of DPYD expression versus TP53WT expression

RSEM-normalized gene expression values for TP53 and DPYD from TCGA colorectal RNA-seq data (COAD + READ) are shown, for subjects with no reported TP53 mutations. The Spearman rank correlation is rho=-0.1501, indicating a downward trend, but is not statistically significant ($p = 0.0523$). The horizontal line segments show the median ($\log_{10}$) expression levels of DPYD in subjects with low (< 1000) and high (> 1000) expression of TP53.

SUPPLEMENTAL TABLE 1

**Region**	**Primers**
**R-0.795**	**F-GACCAAAGCAGATCATTAAGAGG**
	**R-GCAAGACTGCCAAAGGTATGA**
**R-0.820**	**F-AACCCTAGCTCTGCCTCTTG**
	**R-TGGAGTATGTGATGTGCTTCTT**
**R-0.827**	**F-ATACCCACCCAGGCACAA**
	**R-CTCAGAAGAACCCGAGGAGA**
**R-0.829**	**F-ACTGGAAGCGGAGACGAG**
	**R-TCCTGCATGTGAGTGTAGGTG**
**R-0.840**	**F-AACACTCCTTCGTTGCTCGT**
	**R-TGAGGGACATCTGGGTTCTT**
**R-0.891**	**F-CTAAGGTTTTGCTCCCTTGA**
	**R-AGGACAAGTGGTGGCAGTGT**

SUPPLEMENTAL TABLE 2

Number of Patients	DUKE STAGE, N (%)			ADJCTX, N (%)	ADJXRT N, (%)	COX *P*-VALUE	HR [CI95%]
	A	B	C				
N=226	41 (18.1)	94 (41.6)	91 (40)	87 (38.5)	22(9.7)	0.001050	1.68 [1.23 - 2.29]

Supplemental Fig 1

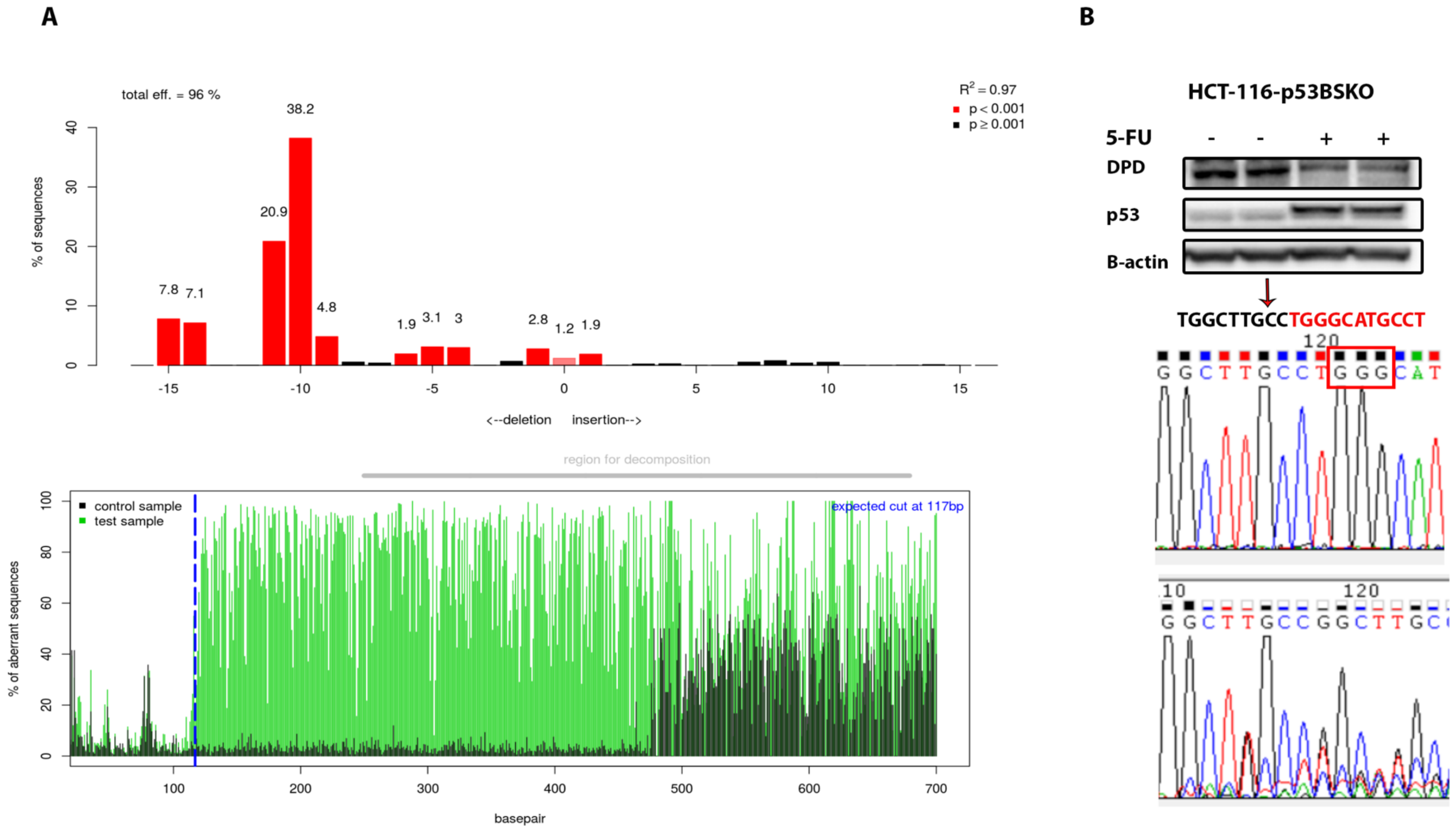

Figure S1: **Analysis of DPYD protein expression in CRISPR edited p53 binding site in HCT-116 cells**. (A) TIDE analysis of depicting the frequency of deletion in the p53BS in HCT-116, Over 78.8% of clones have lost half of the p53 binding sites, Total editing efficiency is 96% (Lower panel) Region of decomposition depicting aberrant nucleotide sequence of edited cells. (green) vs WT cells (Black). (B)

Repression of DPYD following 5-FU (384uM) for 24hrs in p53BSKO HCT-116 cells. (Lower panel) shows the p53 binding site sequence and sequence in red is lost after the cut by CAS9/sgRNA complex indicated by the downward arrow)

Supplemental Fig 2

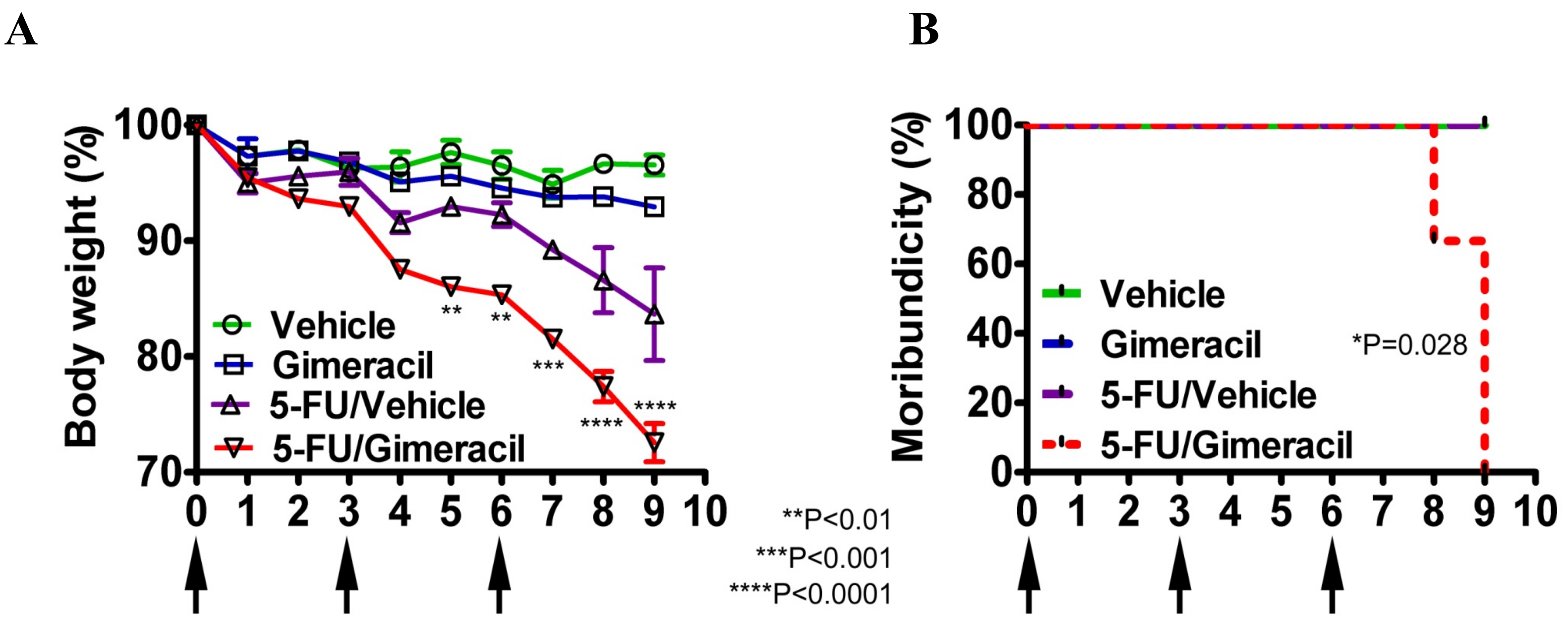

Figure S2: Impact on bodyweight and survival of mice targeted by Gimeracil following IV administration of 5-FU. (A) WT C57BL/6 mice were given vehicle, 5-FU 50mg/kg BW IV or 5-FU and Gimeracil (22.4 mg/kg BW IV) or Gimeracil alone every 3 days over a week. Body weights of these mice were measured every other day and represented as percentage of Day 0. (B) The moribundicity of mice following treatment as in (A). Statistical analysis was carried out using student t-test and P Values are indicated in figures.

Supplemental Fig 3

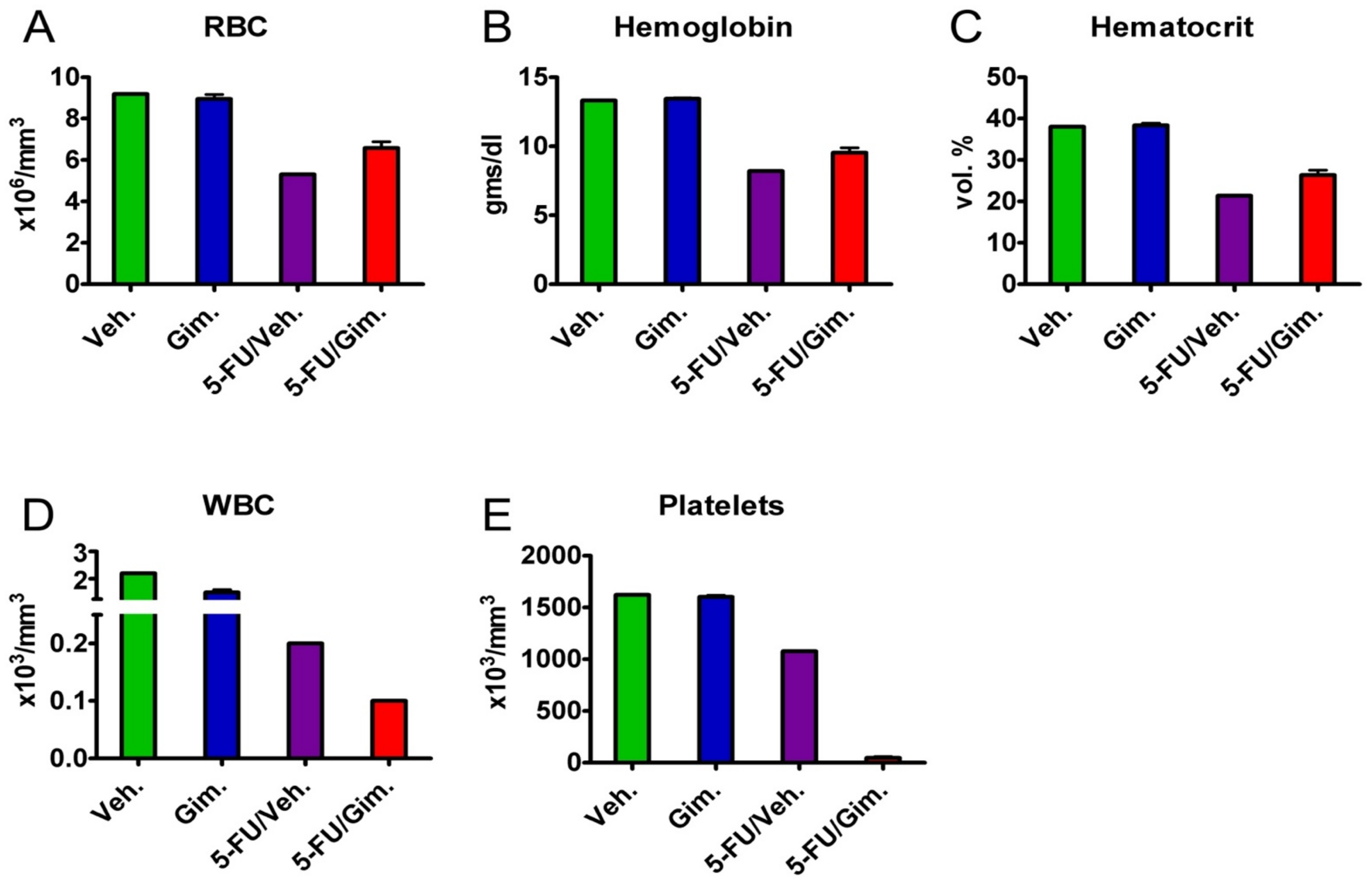

Figure S3: Assessment of Hematological parameters following IV 5-FU administration. CBC analysis following treatment with 5-FU and Gimeracil as treated in Fig S1.

Supplemental Fig 4

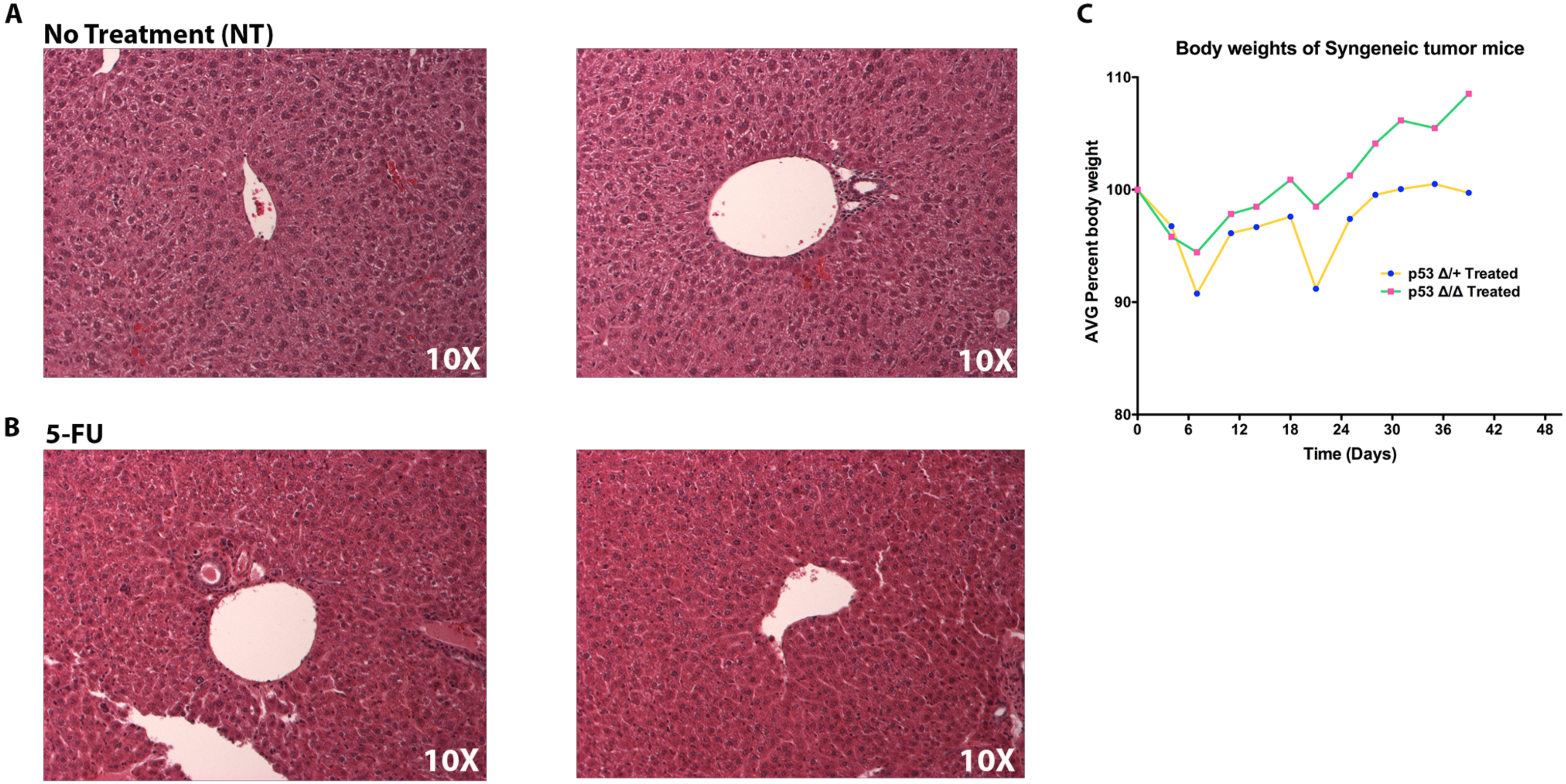

Figure S4: **Assessment for liver toxicity following IV 5-FU treatment of tumor bearing mice**. (A)&(B) H&E Sections of liver from NT (i.e Vehicle) and 5-FU treated (6 weeks) in Albcre;mT/mG;$p53^{\Delta/\Delta}$ mice. (C) Average body weights of mice from syngeneic tumor study.

Supplemental Fig 5

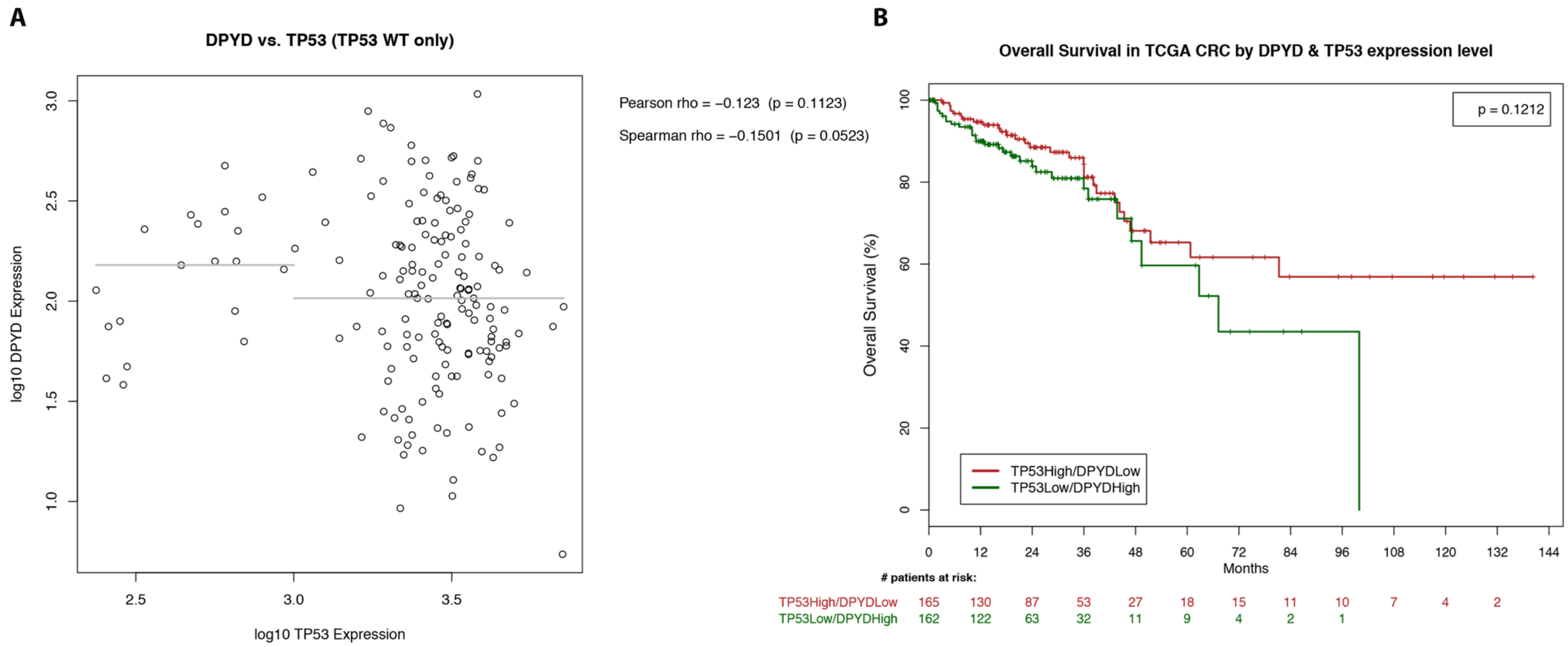

Fig. S5 (A) TCGA analysis of DPYD and p53 expression and correlation with overall survival of CRC patients. RSEM-normalized gene expression values for TP53 and DPYD The Spearman rank correlation is rho=-0.1501, indicating a downward trend, (p = 0.0523). The horizontal line segments show the median (log_{10}) expression levels of DPYD in subjects with low (< 1000) and high (> 1000) expression of TP53. (B) "Kaplan-Meier plot of overall survival in patients with Tp53High/DPYDlow vs Tp53Low/DPYDHigh groups.

Supplementary Fig 6

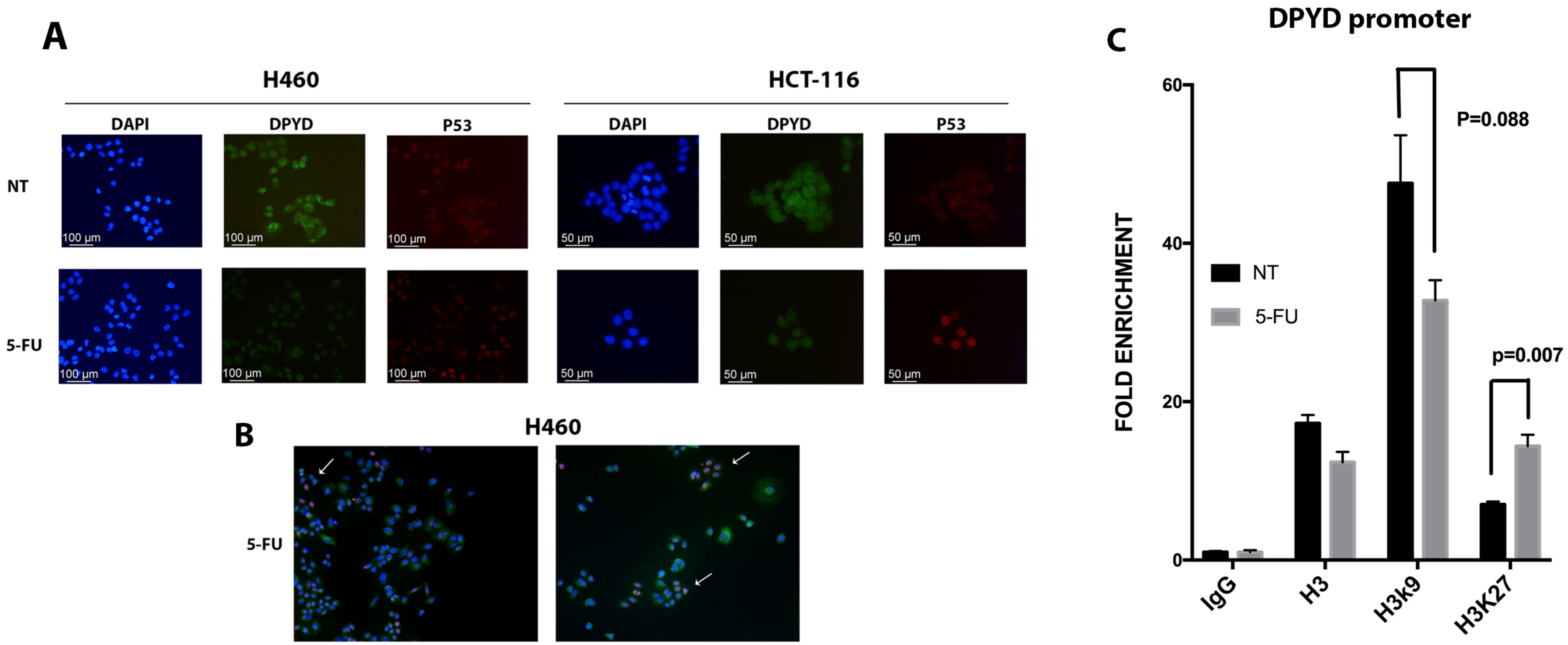

Fig S6: **Immunofluorescence for DPYD and p53 in HCT-116 WT and H460 and H3K9Ac and H3K27me3 enrichment cells treated with either Vehicle or 5-FU(384uM)**. (A) Representative images of HCT-116 and H460 cell show decreased expression of DPYD and increased p53 nuclear staining following administration of 5-FU(384uM) for 24hrs. (B). Merged images of DPYD and p53 showing cells with increased p53 expression and nuclear localization and reduced DPYD expression (see arrows). (C) H3K9Ac and H3K27m3 marks at DPYD promoter in H460 cells following 5-FU(384uM) for 24hrs (n=3) P value calculated by multiple t-test.

Supplementary Fig S7

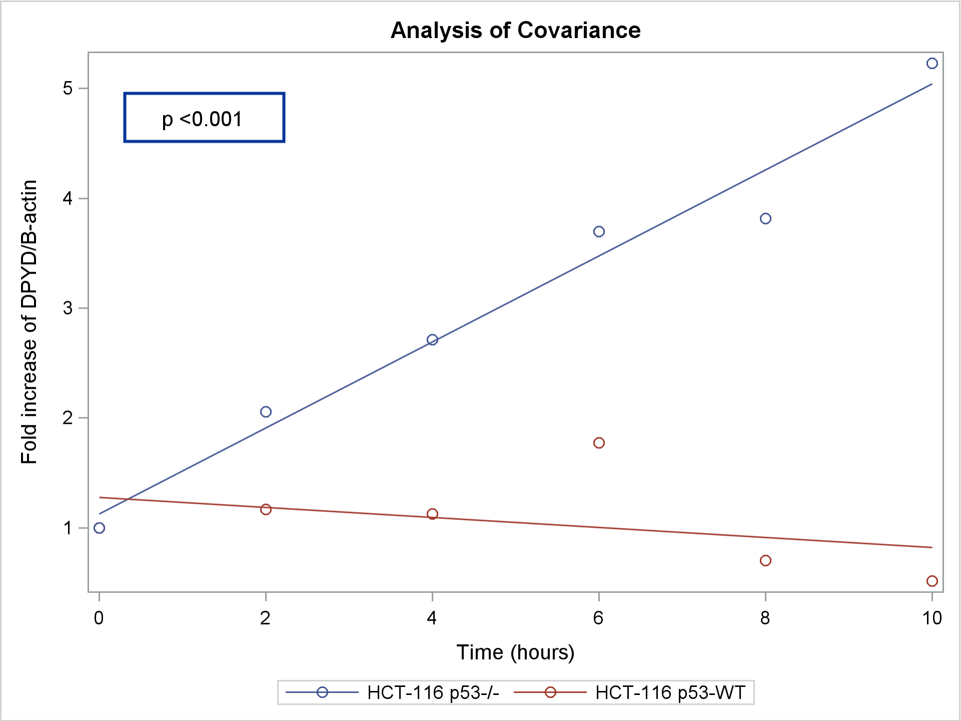

Fig S7: Time course analysis of expression of DPYD protein in HCT-116-WT and HCT-116-p53-/- cells following 5-FU administration: Analysis of covariance results revealed a significant difference in DPYD protein expression over time between HCT-116 WT vs HCT-116p53-/- ($p < 0.001$). The 12-hour time point was excluded from both groups in the analysis due to technical error in assessing protein expression in HCT-116 p53-/- group.

Supplementary Fig 8 original blots

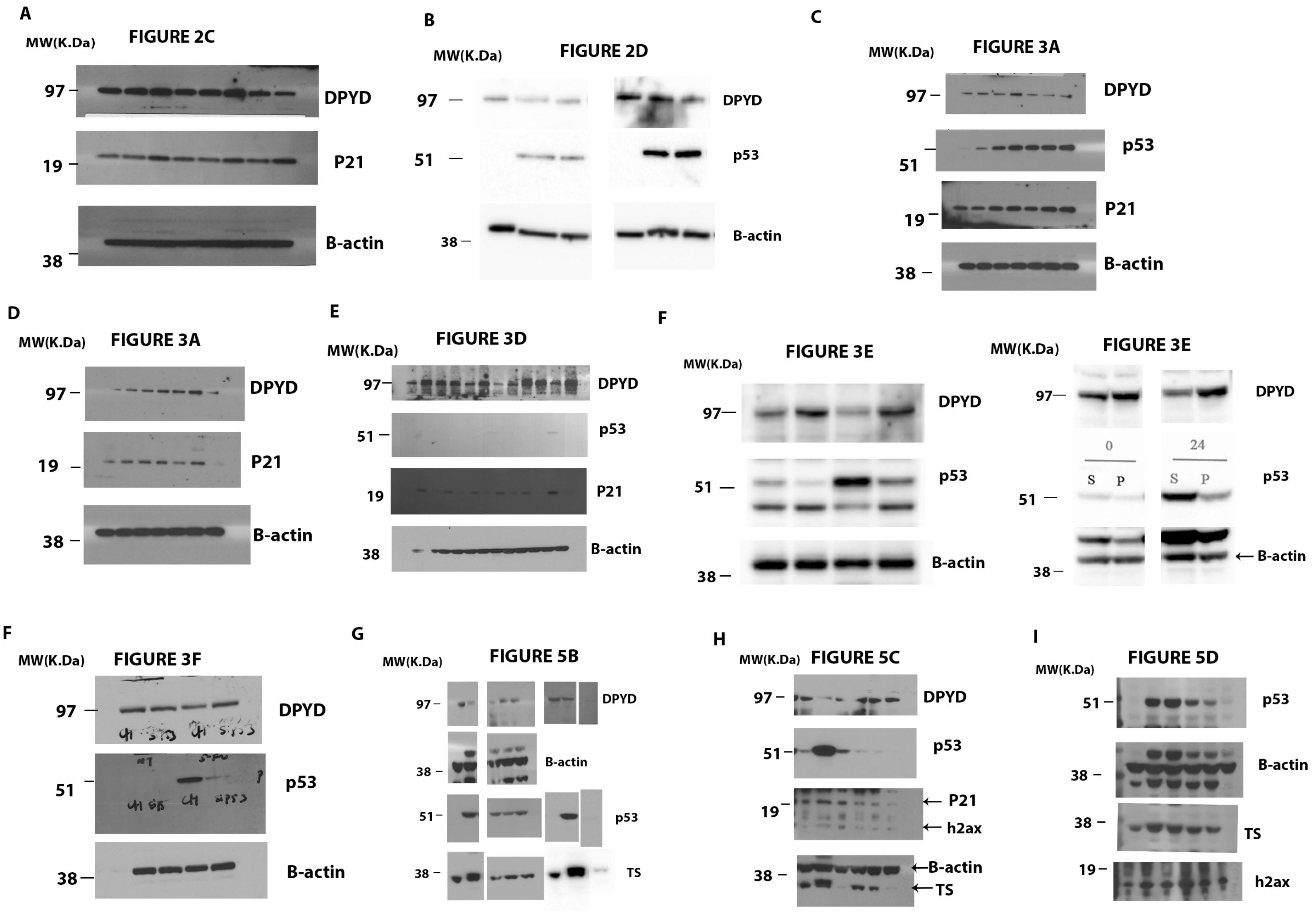

Supplementary Fig 8 original blots continued

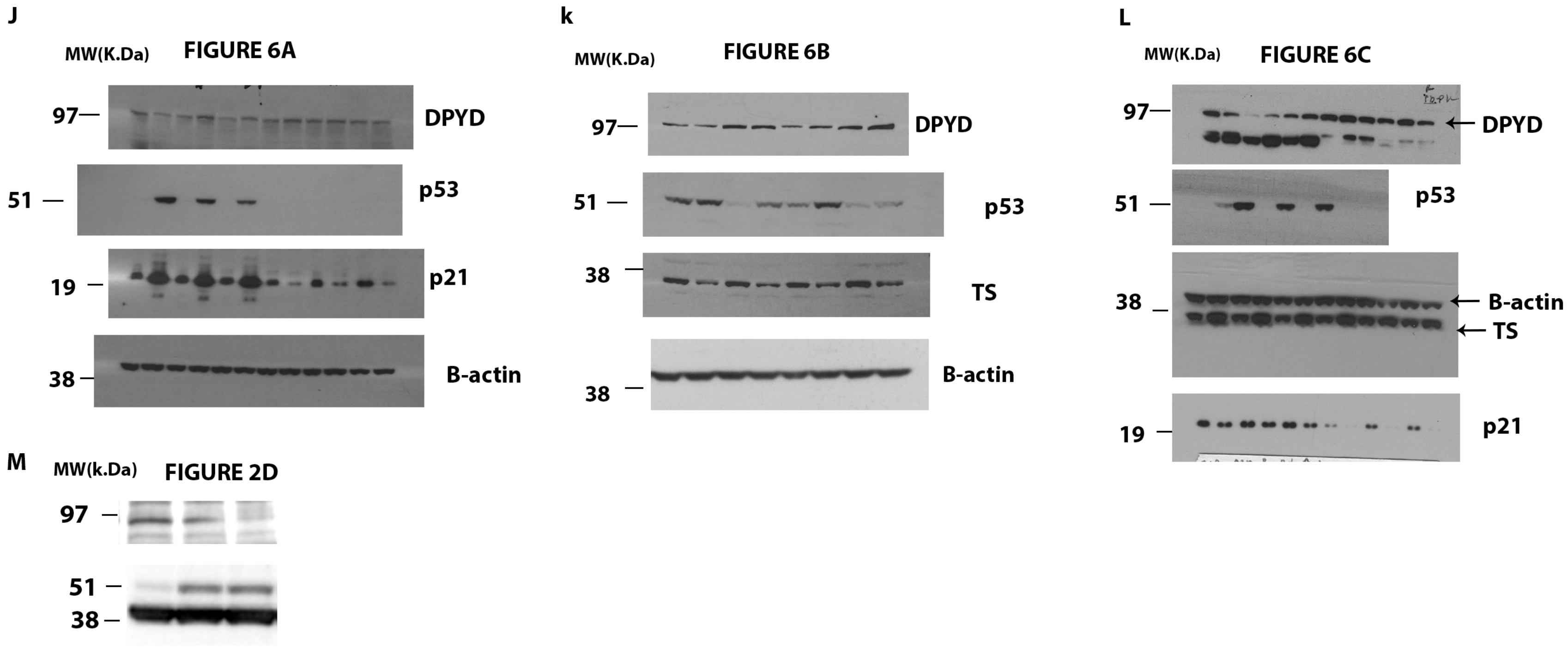

Fig S6: Original western blotting images. The figures in the main text corresponding to blots are indicated on top of the blot**.**
